# Interplay of precision therapeutics and MD study: *Calocybe indica*'s potentials against cervical cancer and its interaction with VEGF via octadecanoic acid

**DOI:** 10.1111/jcmm.18302

**Published:** 2024-04-23

**Authors:** Suhana Datta, Preeti Verma, Bikram Dhara, Rita Kundu, Swastika Maitra, Arup Kumar Mitra, Mohd Shahnawaz Khan, Torki A. Zughaibi, Shams Tabrez, Ajoy Kumer

**Affiliations:** ^1^ Department of Microbiology St. Xavier's College Kolkata West Bengal India; ^2^ Department of Botany, Centre of Advanced Studies University of Calcutta Kolkata West Bengal India; ^3^ Department of Health Sciences Novel Global Community Educational Foundation Habersham, NSW Sydney Australia; ^4^ Center for Global Health Research, Saveetha Medical College and Hospital Saveetha Institute of Medical and Technical Sciences Chennai India; ^5^ Department of Biochemistry, College of Science King Saud University Riyadh Saudi Arabia; ^6^ King Fahd Medical Research Center King Abdulaziz University Jeddah Saudi Arabia; ^7^ Department of Medical Laboratory Sciences, Faculty of Applied Medical Sciences King Abdulaziz University Jeddah Saudi Arabia; ^8^ Department of Chemistry, Department of Chemistry, College of Arts and Sciences IUBAT‐International University of Business Agriculture & Technology Dhaka Bangladesh

**Keywords:** *Calocybe indica* P&C, cervical cancer, drug delivery, exosomes, nanotherapeutics, natural products, precision therapeutics, Protacs, small molecules

## Abstract

The evolving landscape of personalized medicine necessitates a shift from traditional therapeutic interventions towards precision‐driven approaches. Embracing this paradigm, our research probes the therapeutic efficacy of the aqueous crude extract (ACE) of *Calocybe indica* in cervical cancer treatment, merging botanical insights with advanced molecular research. We observed that ACE exerts significant influences on nuclear morphology and cell cycle modulation, further inducing early apoptosis and showcasing prebiotic attributes. Characterization of ACE have identified several phytochemicals including significant presence of octadeconoic acid. Simultaneously, utilizing advanced Molecular Dynamics (MD) simulations, we deciphered the intricate molecular interactions between Vascular Endothelial Growth Factor (VEGF) and Octadecanoic acid to establish *C.indi*ca's role as an anticancer agent. Our study delineates Octadecanoic acid's potential as a robust binding partner for VEGF, with comprehensive analyses from RMSD and RMSF profiles highlighting the stability and adaptability of the protein–ligand interactions. Further in‐depth thermodynamic explorations via MM‐GBSA calculations reveal the binding landscape of the VEGF–Octadecanoic acid complex. Emerging therapeutic innovations, encompassing proteolysis‐targeting chimeras (PROTACs) and avant‐garde nanocarriers, are discussed in the context of their synergy with compounds like *Calocybe indica* P&C. This convergence underscores the profound therapeutic potential awaiting clinical exploration. This study offers a holistic perspective on the promising therapeutic avenues facilitated by *C. indica* against cervical cancer, intricately woven with advanced molecular interactions and the prospective integration of precision therapeutics in modern oncology.

## 1 INTRODUCTION

The profound challenge posed by cancer, characterized by its relentless spread and malignancy, has positioned it as a paramount health concern. Particularly worrying are its various forms such as lung, colorectal, stomach, liver and breast cancers, which continue to exact a grim toll on human lives.^1^ Among these, cervical cancer, predominantly caused by the Human Papillomavirus (HPV), is especially prevalent in low‐ and middle‐income countries, necessitating an aggressive research and intervention stance.^2^, ^3^, ^4^, ^5^, ^6^ Recognizing this urgency, the International Agency for Research on Cancer (IARC) has themed 2023 as ‘Ending cervical cancer within a few generations’, thereby emphasizing the essentiality of innovation in interventions, spanning from treatment regimens to HPV vaccination awareness.^7^, ^8^ Current challenges in cancer treatment methodologies, including their economic burden and associated adverse reactions, have opened the doors for exploration in Complementary Alternative Medicine (CAM).^9^, ^10^ Natural extracts, especially those from mushrooms, emerge as potential therapeutic agents, with *Calocybe indica* P&C, rich in nutritional value, being traditionally recognized for its medicinal properties.^11^, ^12^, ^13^, ^14^, ^15^ This mushroom variety is low in calorie and fat, rich in protein, carbohydrate, minerals and a significant amount of essential amino acids. Studies have been done on its nutraceuticals, antioxidant, anticancer, prebiotic, immunomodulatory, anti‐inflammatory, antimicrobial, reduction of hypercholesterol and antidiabetic activities till date. The fruitbodies of *C.india* were grown on paddy straw and sugarcane bagasse substrate combination (1:1) with garden soil: VC: sand (1:1:0.5) casing. The age of the sporocarp during harvesting was 35–40 days.

Current research is the first attempt to study the in vitro effect of *C.indica* fruitbodies extracted in different solvents on cervical cancer cell line SiHa (HPV 16+) along with its underlying mechanism and identification of bioactive compounds by bioassay guided fractionation. It was also aimed to investigate the *C. indica* fruitbody extracts to stimulate the growth of probiotic bacteria like *Lactobacillus* spp. and its potential to be exploited as a prebiotic agent.

Navigating the complexities of clinical practice, the prime directive has always been to enhance treatment efficacy while mitigating adverse effects. Precision therapeutics has emerged as a beacon in this context, propelled by advancements such as genetic sequencing and molecularly targeted drug exploitation.^16^ Unlike the conventional ‘one‐size‐fits‐all’ methodology, precision therapeutics aims to provide bespoke treatments tailored to individual patients, utilizing a broad spectrum of data, ranging from genomes to digital health measurements. This focus on theragnostic (diagnostic and treatment) approaches encapsulates the evolving landscape of clinical practice. The implications of such methodologies are vast, influencing treatment modalities across cancers, neurological conditions and inflammatory diseases. Landmark developments in this realm include the discovery of novel small molecules, the synthesis of ubiquitination‐mediated protein degradation by proteolysis‐targeting chimeras (PROTACs), and innovations in mRNA‐targeted therapies.^17^, ^18^ The pivot to precision therapeutics has heralded a complementary shift in drug delivery techniques, prominently featuring nanocarriers in the field of nanomedicine. The utilization of exosomes and liposomes to ferry precision therapeutic agents, backed by recent studies evidencing their efficacy without overt toxicities, exemplifies the evolution of drug delivery methods.

Our study carves out a unique niche by merging traditional botanical insights with state‐of‐the‐art scientific methodologies. The exploration of *C. indica's* potential in cervical cancer therapeutics, combined with the insights into precision therapeutics, aims to forge a renewed path in oncology. Beyond just scientific rigour, our study embodies an unwavering commitment to human health, signifying hope in the face of adversity.

## 2 MATERIALS AND METHODS

### 2.1 Molecular docking

The objective of this study was to elucidate the binding affinity and interaction patterns between the receptor protein PDB ID: 1VPP, and the active compounds derived from *C. indica*. Our investigative approach sought to harness the power of computational methodologies to forecast octadecanoic acid‐receptor binding efficiency. Central to our methodological repertoire was molecular docking, a powerful predictive technique. The Autodock version 4.2.6 software, esteemed for its precision in molecular docking simulations, was our computational tool of choice.^19^, ^20^ An imperative initial step was the meticulous pre‐processing of both the 1VPP receptor and the active compounds. This encompassed the amalgamation of non‐polar hydrogens with their parent molecules, with subsequent conversion and storage of the optimized structures in .pdbqt format, a format compatible with Autodock simulations. A pivotal aspect of molecular docking pertains to the accurate demarcation of the octadecanoic acid‐binding region on the receptor. In our study, this was achieved through the use of grid boxes, the dimensions of which were scrupulously determined, taking into account the likely interaction site on 1VPP. Furthermore, a grid spacing of 0.3 Å was adopted to strike a judicious balance between computational feasibility and simulation accuracy. For the purpose of searching and sampling, the expansive conformational space inherent to protein–octadecanoic acid interactions, we selected the Lamarckian Genetic Algorithm (LGA). Recognized for its efficacy in such endeavours, the LGA served as our preferred search paradigm. To bolster the validity and robustness of our findings, we executed three distinct molecular docking runs. Within each run, we aimed for 50 solutions, commencing with a diverse initial population size set at 500. We stipulated a maximum of 2,500,000 evaluations, with the generational ceiling set at 27, to guarantee a satisfactory convergence of the solutions. Barring these adjustments, default Autodock parameters were adhered to, aligning our methodology with established norms in the field. Post‐docking, the imperative task of solution evaluation took centre stage. For this, we employed RMSD clustering, a technique proficient in categorizing similar docking poses by their spatial alignments. To further refine our analysis, the clusters underwent a re‐assessment, adopting a clustering tolerance of 2.0 Å. The dominant cluster, characterized by its minimal energy score and maximal population, was earmarked as the most plausible binding configuration of the *C. indica* active compounds with 1VPP. This methodical and rigorous approach, underpinned by computational excellence, has bestowed upon us a deeper insight into the intricate dance of interactions between 1VPP and the active compounds of *C. indica*, propelling us closer to potential therapeutic ventures or enriched molecular explorations.^19^, ^20^, ^21^

### 2.2 Molecular dynamics simulation (MDS)

**FIGURE 1:**
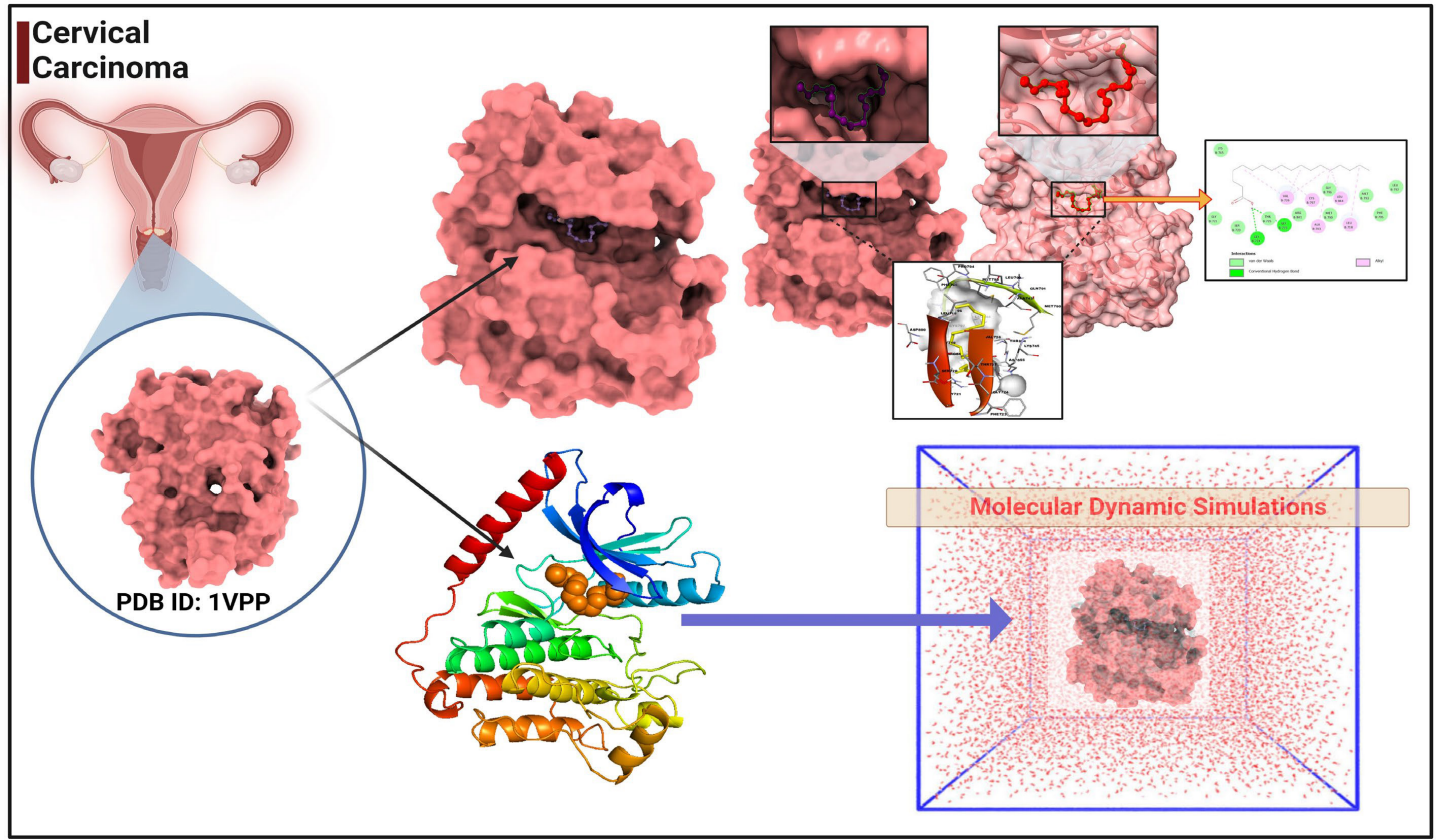
Overview of molecular docking and molecular dynamic simulation.

The MD simulations were conducted to examine stability of the complexes formed by 1VPP and active compounds of *C. indica*. The simulations were performed using the Desmond 2020.1 software.^22^, ^23^ Independent simulations were carried out for each complex, including the control, at a temperature of 27°C. To represent the molecular interactions, the OPLS‐2005 force field was employed, which has been described in previous studies.^24^, ^25^, ^26^ An explicit solvent model was used, utilizing SPC molecules.^27^, ^28^ The simulation system OCTADECANOIC ACIDs enclosed in a periodic boundary salvation box with dimensions of 10 Å × 10 Å × 10 Å. Sodium ions (Na^+^) were added to neutralize the system's charge, and NaCl solutions were introduced to mimic the physiological environment. The equilibration process involved an NVT ensemble simulation for 10 ns to allow the protein–octadecanoic acid complexes to stabilize. This step was performed to ensure that the system reached a steady state.^29^, ^30^ Following this, a short equilibration and minimization phase were carried out using an NPT ensemble for 12 ns. The NPT ensemble was set up using the Nose–Hoover chain coupling scheme,^31^ with a varying temperature and relaxation time of 1.0 ps. The pressure was maintained at 1 bar throughout all the simulations. A time step of 2 fs was used for the simulations. Pressure control OCTADECANOIC ACIDs achieved using the Martyna–Tuckerman–Klein chain coupling scheme,^31^, ^32^ with a relaxation time of 2 ps. The particle mesh method is employed to calculate long‐range electrostatic interactions,^31^ with a Coulomb interaction radius fixed at 9 Å. The RESPA integrator was utilized with a time step of 2 fs for each trajectory to calculate the bonded forces. Subsequently, a final production run of 100 ns was performed for each simulation. To assess the stability of the MD simulations, various parameters were calculated, including the root mean square deviation (RMSD), radius of gyration (Rg), root mean square fluctuation (RMSF) and the number of hydrogen bonds (H‐bonds). These measures were employed to monitor the stability of the system throughout the simulations (Figure 1).

### 2.3 Binding free energy analysis

The assessment of the binding free energies of protein–octadecanoic acid complexes is pivotal in understanding their stability and binding affinities. For this investigation, the state‐of‐the‐art method, Molecular Mechanics combined with the Generalized Born and Surface Area (MM‐GBSA) approach, was adopted.^33^ The MM‐GBSA methodology is a widely accepted computational technique that offers insights into the thermodynamics of protein–OCTADECANOIC ACID interactions. This approach combines the rigour of molecular mechanics force fields with implicit solvent models, effectively balancing computational efficiency and accuracy. To ensure precision in our study, the Prime MM‐GBSA binding free energy calculations were executed using a dedicated Python script titled ‘thermal_mmgbsa.py’. This script was specifically designed to process the molecular dynamics (MD) simulation trajectories and extract relevant energy data. The trajectory analysis was confined to the last 50 frames of the MD simulations, ensuring that the system had likely achieved equilibrium and the resultant interactions were representative of stable protein‐octadecanoic acid complexes. With a sampling rate of one frame per step, the data extracted were both comprehensive and pertinent to the binding event. Applying the principle of additivity, as described by,^34^ the MM‐GBSA binding free energy (expressed in kcal/mol) was calculated. This foundational principle entails the aggregation of distinct energy components that dictate the interaction between the octadecanoic acids and protein. These components encompass:

- Coulombic interactions—electrostatic forces between charged entities.
- Covalent bonds—the energies associated with bond formation.
- Hydrogen bonds—specific non‐covalent interactions crucial for molecular recognition.
- Van der Waals forces—dispersion forces contributing to the stability of the complex.
- Self‐contacts—interactions within a single molecular entity.
- Lipophilic interactions—hydrophobic forces that play a role in octadecanoic acid binding.
- Solvation energies—these represent the energetic cost or gain when the protein and octadecanoic acid move from a solvated to a bound state.

By systematically evaluating these individual energy contributions, a holistic understanding of the binding thermodynamics of the protein–octadecanoic acid complex was achieved. This detailed analysis not only offers insights into the stability of the complex but also provides valuable data for potential drug design endeavours and further biophysical investigations.

The equation used to calculate Δ*G*_bind_ is as follows:

$$\Delta G_{\text{bind}}=\Delta G_{\text{Covalent}}+\Delta G_{\text{Hbond}}+\Delta G_{\mathrm{vdW}}+\Delta G_{\text{Lipo}}+\Delta G_{\text{Solv}\_\mathrm{GB}}$$

In this equation, each term represents the contribution of a specific energy component to the overall binding free energy (Δ*G*_bind_). The MM‐GBSA approach allows for a comprehensive estimation of the binding affinity by considering multiple energetic factors.

### 2.4 Fruiting body collection and procedure for solvent extraction

Fruiting bodies of *C. indica* were meticulously sourced from a specialized mushroom cultivation facility within the precincts of Ramakrishna Mission (RKM) Narendrapur, located in West Bengal. The specimens underwent a rigorous identification process, cross‐referenced with authoritative literature to ensure precision. The whole fruitbody of *C.indica* was used for extraction.

The extraction procedure of *C. indica* was executed with scientific precision using 100 g of desiccated fruiting bodies. The protocol involved multiple solvent systems to ensure comprehensive extraction of bioactive components. The specific methodology entailed:

*Chloroform extraction*: The botanical material was treated with 500 mL of analytical‐grade chloroform, yielding the designated chloroform extract, labelled as CE.
*Methanolic extraction*: Subsequently, methanol was employed as a solvent and the methanolic crude extract, denoted as MCE, was procured.
*Aqueous extraction*: The residual matrix was subjected to boiling water (maintained at 100°C for 3 h), leading to the acquisition of the aqueous crude extract, referred to as ACE.

Post‐extraction purification and concentration steps were scrupulously implemented:

- The CE and MCE extracts were filtered through premium Whatmann filter paper No. 1, ensuring the elimination of particulate matter.
- These filtrates were then concentrated under reduced pressure using a state‐of‐the‐art rotary vacuum evaporator.
- The aqueous‐crude extract ACE, underwent a lyophilization process, converting them into a stable, dry form.

To safeguard against potential degradation and to preserve the bioactive integrity of the compounds, all extracted preparations were securely stored under controlled conditions at −20°C. This methodological rigour ensures the extracts are of optimal quality for subsequent analytical and experimental endeavours.

### 2.5 Detailed evaluation of anticancer properties of *C. indica*

#### 2.5.1 Cellular cultivation and maintenance

The SiHa human cervical cancer cell line, exhibiting the HPV16+ profile, was graciously sourced from the National Center for Cell Science (NCCS), Pune. These cells were cultured in a nutrient‐rich milieu, provided by Dulbecco's Modified Minimum Essential Medium (DMEM) from Himedia. This medium was supplemented with 10% fetal bovine serum and a meticulously calibrated antibiotic–antifungal cocktail, ensuring both nutrition and aseptic conditions for the cells. A controlled environment, set at 37°C with a pH of 7.4 and a consistent 5% CO_2_ atmosphere, was deemed optimal. Depending on the specific experimental requirements, cells found themselves housed in various vessels, such as T25 flasks, 60‐mm Petri dishes, and cover slips treated with Poly‐l‐Lysine. Subcultures and cell division were performed methodically, using a solution containing 0.25% trypsin and 0.02% EDTA. Alongside, PBMCs derived from healthy volunteer blood samples were cultivated in the Roswell Park Memorial Institute‐1640 medium (RPMI‐1640), serving as an essential reference for assessing cytotoxicity.

#### 2.5.2 MTT assay

To meticulously gauge the post‐treatment vitality of the SiHa cell line, the MTT reduction method was chosen due to its established credibility. Extracts from *C. indica* were methodically prepared in a concentration series ranging from 0 to 600 μg/mL. SiHa cells, as well as isolated PBMCs, were cultured and post an initial incubation spell, were exposed to this concentration gradient. After a stipulated duration, the medium was replaced with the MTT solution. Post a 4‐h incubation, formazans crystals formed were solubilized and the optical density was captured at 595 nm wavelength using a BioRad microplate reader.

#### 2.5.3 Clonogenic assay

This assay's underpinning was the exploration of a cell's inherent ability to proliferate and form colonies. Post the seeding process, and an overnight incubation, the SiHa cells were introduced to the IC_50_ concentration of ACE. This was followed by a seven‐day incubation period. Post this phase, the grown colonies underwent fixation and staining. The stained colonies were digitized and counted using Image J software. Throughout this assay, Doxorubicin (1 μM) was employed as a standard control.

#### 2.5.4 Cellular migration via wound healing assay

Simulating a wound or injury, a sterile pipette tip was used to create a linear scratch across a confluent monolayer of SiHa cells. After this ‘wounding’, cells were treated with the IC_50_ concentration of ACE. The ensuing migration and wound‐closure process of these cells were diligently captured at both the initial and 24‐h marks, with the help of a high‐resolution inverted microscope from Leica.

#### 2.5.5 Insight into nuclear architecture changes

Post their overnight incubation, SiHa cells, which had been seeded on poly‐L‐lysine coated coverslips, were treated with the IC50 dose of ACE. After a subsequent incubation phase, these cells underwent a fixing process with para‐formaldehyde, ensuring their structures remained intact for further analysis. This was followed by staining with the Hoechst 33258 dye, a renowned nuclear stain. An advanced fluorescent microscope was then employed to observe any nuclear morphological deviations. Furthermore, digital tools such as Fiji/Image J software were employed to quantify aspects such as nuclear shape changes, fluorescence intensity variance, and other nuclear parameters, providing an in‐depth understanding of the treatment's effect at the cellular nucleus level.

#### 2.5.6 Assessment of intracellular reactive oxygen species (ROS) production

In the intricate realm of cellular biology, the production of reactive oxygen species (ROS) serves as an important marker for evaluating the potential cellular damage and redox state. For this study, we meticulously monitored ROS generation in the SiHa (HPV 16+) cell line, standardized at a concentration of 1X10^6^ cells/ml. The cells were exposed to the IC_50_ concentration of ACE. For comparison, 1 mM N‐acetyl cysteine (NAC), a renowned antioxidant, served as the negative control, while 100 μM H_2_O_2_, an established inducer of ROS, functioned as the positive control. Following a 3‐h incubation, cells were twice washed with phosphate‐buffered saline (PBS) and subsequently resuspended in a solution containing CM‐H2DCFDA (5 μM), a recognized probe for ROS detection. Ensuring the maintenance of a controlled environment, the resuspension was protected from light and incubated for 30 minutes. Flow cytometry analyses were then executed using the state‐of‐the‐art BD FACS Verse equipment.

#### 2.5.7 Detailed cell cycle progression analysis

Understanding the cellular response post‐exposure to external agents often necessitates a thorough evaluation of cell cycle dynamics. In this context, SiHa cells (density: 5 × 10^5^) were treated with the IC_50_ dosage of ACE. After incubation for a full 24‐h cycle under standard conditions (37°C with a 5% CO_2_ atmosphere), these cells were then fixed using 70% ethanol, ensuring preservation of cellular architecture and subsequently stored at a crisp 4°C, awaiting further examination. Pre‐analysis procedures incorporated a PBS rinse, followed by the addition of DNase‐free RNase to eliminate any traces of RNA—an incubation that lasted for 2 h at 37°C. The final step involved staining the cells with propidium iodide (PI), a reliable DNA intercalating agent. This facilitated an accurate assessment of DNA content via the BD FACS Verse flow cytometer. Data processed through the Cell Quest Pro software allowed a comprehensive understanding of the proportion of cells distributed across different cell cycle phases.

#### 2.5.8 Examination of apoptotic indices using annexin V‐FITC/PI double staining

Apoptosis, an orchestrated cellular suicide mechanism, has often been a focus of oncological studies. In this light, SiHa cells (5 × 10^5^) were cultivated overnight and subsequently exposed to the IC50 concentration of ACE. Following a 24‐h period in a controlled environment (37°C, 5% CO_2_), these cells were then harvested, and twice washed with 1× PBS. The staining mechanism, performed in a light‐shielded setting, utilized Annexin V‐FITC, which specifically binds externalized phosphatidylserine, and propidium iodide for nucleic acid visualization. Adherence to the meticulous protocol furnished by BD Biosciences ensured robust results. The culmination of this process saw these stained cells processed through the BD FACS Verse flow cytometer, effectively categorizing them into distinguishable viable, early apoptotic, late apoptotic and necrotic segments.

With this rigorous methodology, the depth and breadth of our exploration into ACE's effects on SiHa cells stand fortified, promising both reliability and scientific insight.

#### 2.5.9 Bacterial strain acquisition and propagation

From the distinguished P.G. Department of Microbiology at St. Xavier's College, Kolkata, three probiotic strains, notably *Lactobacillus plantarum* ATCC 4356, *Lactobacillus rhamnosus* ATCC 7469, and *Lactobacillus acidophilus*, were meticulously procured in their pristine pure culture form. Recognizing their critical contribution to maintaining a balanced gut microenvironment, these strains were cultured under highly controlled conditions. MRS broth and agar media, renowned for supporting lactobacilli growth, were employed, and a consistent temperature of 37°C was maintained, mirroring the gut's natural warmth.

#### 2.5.10 Systematic extraction of aqueous crude polysaccharides (ACP)

*Calocybe indica's* aqueous crude extract (ACE) was subjected to a polysaccharide extraction process. A deliberate threefold volume of ethanol was added to promote precipitation of the desired components. Following an overnight stabilization at a cool 4°C, centrifugation was applied at a significant 10,000 rpm force for 15 min, ensuring the extraction of the polysaccharide mixture termed ACP. To enhance purity, the collected precipitate underwent sequential washes with ethanol, ethyl acetate and acetone. A lyophilization process subsequently rendered the ACP into a stable, storage‐friendly form.

#### 2.5.11 Probiotic bacterial interaction with ACP

For this phase, the strains *L. plantarum*, *L. rhamnosus* and *L. acidophilus* were cultivated in lactobacilli‐optimized MRS broth for a period of 12 h at the consistent temperature of 37°C. After this growth phase, a volume of 100 μL from the bacterial log culture was precisely introduced into sterilized 10 mL MRS broth. For comparative analysis, a control set‐up containing 1.5% dextrose and maltodextrin was prepared. The experimental set‐up was enriched with 1.5% ACP. Over the subsequent 48 h, absorbance was closely monitored at 600 nm at pre‐decided intervals, allowing for a comprehensive growth curve mapping. The changing pH values, vital indicators of fermentation dynamics, were captured using a state‐of‐the‐art pH meter.

#### 2.5.12 Acid digestibility of polysaccharides

Simulating the harsh acidic environment of the human stomach, hydrochloric acid buffers at pH values of 1 and 5 were prepared. The main objective was to evaluate the robustness and resilience of the *C. indica* polysaccharides against digestive processes. A 1% weight/volume polysaccharide solution was mixed in a 1:1 ratio with the buffer. Controls were set using maltodextrin and inulin. After 37°C incubation for 2 h, sugar release patterns were analysed using both the dinitro salicylic acid (DNS) assay and the anthrone method. Mathematical models helped determine the polysaccharides' relative digestibility.

#### 2.5.13 Comprehensive HR‐LCMS analysis

To delve deeper into the molecular intricacies of ACE, a sophisticated HR‐LCMS analysis was conducted. The Agilent G6550A model, known for its precision, was employed. Chemical fingerprints of ACE were generated, with acquisition parameters meticulously set in the MS‐ mode, covering a mass range from 130 to 1000 daltons (*M*/*Z*). For the gas chromatography segment, a temperature of 250°C was maintained with a steady flow rate, ensuring compound stability and clarity of results.

#### 2.5.14 Detailed polysaccharide characterization protocols

The procured ACP underwent a multi‐step characterization. Initially, a silica column was used for chromatographic separation. Post‐elution, TLC silica gel played a pivotal role in further fractionation. Visualization was enhanced by the Lieberman‐Burchard reagent. Subsequent pooling and filtration of similar fractions readied the samples for advanced spectrometric analysis.

#### 2.5.15 Advanced spectroscopic and crystallographic techniques

The isolated compounds were introduced to a suite of high‐end analytical instruments housed at Indian Institute of Chemical Biology (IICB) and National Institute of Pharmaceutical Engineering and Research (NIPER), Kolkata. Employing nuclear magnetic resonance, mass spectrophotometry, and X‐Ray crystallography, each compound's unique molecular signature was mapped.

#### 2.5.16 Rigorous statistical approaches

Data processing was undertaken with the GraphPad Prism software (version 5), a gold standard in the field. Any value below a *p*‐value of 0.05 was flagged for its significance. Ensuring the reliability of findings, all presented data stemmed from triplicate measures and were articulated as mean ± standard deviation and significance was evaluated by one way analysis of variance (ANOVA) using prism 5 GraphPad software. The dose dependent effect of ACE and other treatments on the inhibition of SiHa (HPV16+) was determined by linear regression. This comprehensive study, through its methodical approach, seeks to provide unparalleled insights into the multifaceted interactions between *C. indica* extracts and vital gut probiotics.

## 3 RESULTS AND ANALYSIS

### 3.1 Molecular screening and re‐docking

Embarking on a mission to unveil innovative therapeutic applications, we focussed on repurposing certain antidiabetic drugs. Our specific interest lay in targeting the VEGF (PDB ID: 1VPP), a molecule that has garnered attention due to its potential associations with cervical cancer. Central to our exploration is the technique of ‘molecular docking’, a precise computational approach that elucidates the interaction dynamics between two molecules. To this end, we deployed high‐fidelity computer simulations. The aim was to gauge the binding propensities of a suite of phyto compounds from *C. indica*, against cervical cancer target. A comprehensive delineation of these molecular interactions, based on binding affinities and spatial configurations, has bein Table 1. From the results 1VPP and octadecanoic acid possessed a higher binding affinity of −10.17 kcal/mol.

**TABLE 1:** Octadecanoic acids with the most auspicious binding affinity with VEGF (PDB ID: 1VPP) was calculated by molecular docking analysis.

Ligands	Binding affinity (kcal/mol)
Meradimate	−4.10
3‐amino‐2‐naptholic acid	−6.20
Altretamine	−5.60
Proadifen	−7.22
**Octadecanoic acid**	**−10.17**

*Note:* Octadecanoic acid showed highest binding affinity which was a significant result.

**FIGURE 2:**
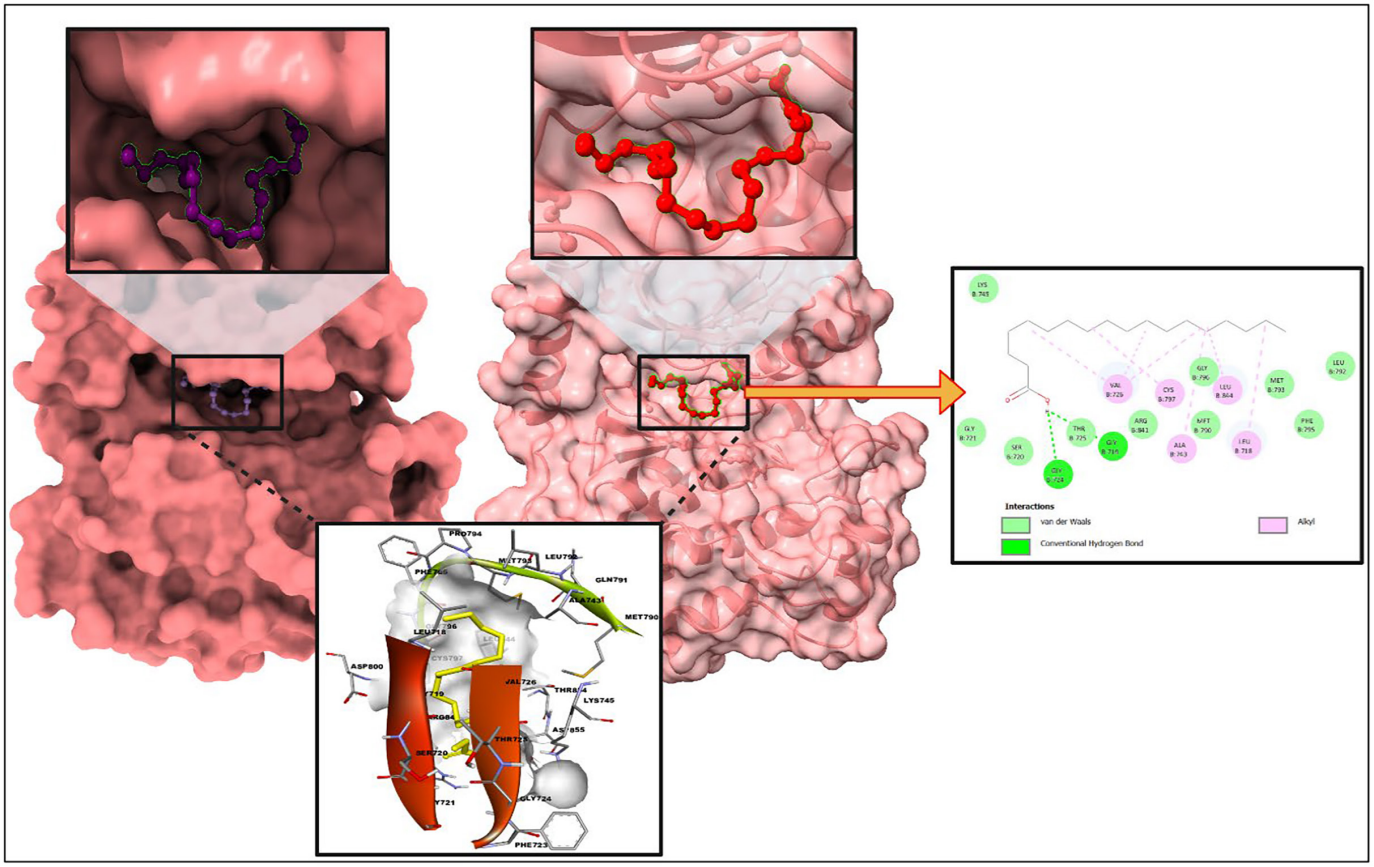
Molecular docking of 1VPP and octadecanoic acid (from *C. indica*) and the dock pose and 2D interaction diagram at lower panels.

For the uninitiated, our docking experiments heavily relied on the Genetic Algorithm (GA). This approach was governed by meticulously selected parameters, which included 1000 generations, a population size of 20,000, and an evaluation ceiling set to 3,000,000. As we delved deeper into the docking dynamics, we integrated the Lamarckian Genetic Algorithm (LGA) to sieve out the protein‐octadecanoic acid complex that boasted the lowest binding free energy (dG)—a proxy for optimal binding dynamics. Such characteristics hint at the duo's potential as formidable agents in the battle against cervical cancer. For a more granular insight, we direct readers to Figure 2, where the docked configurations, juxtaposed with intricate 2D structures of the complexes, are graphically presented.

### 3.2 Molecular dynamics simulation (MDS)

**FIGURE 3:**
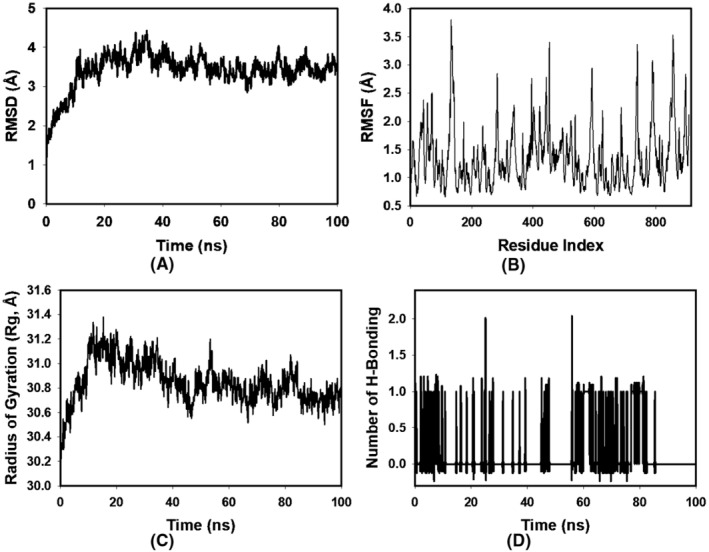
MD simulation analysis of 100 ns trajectories of (A) Cα backbone RMSD of 1VPP + OCTADECANOIC ACID; (B) RMSF of Cα backbone of 1VPP + OCTADECANOIC ACID; (C) Cα backbone radius of gyration (Rg) of 1VPP + OCTADECANOIC ACID; (D) Formation of hydrogen bonds in 1VPP + OCTADECANOIC ACID.

Molecular Dynamics (MD) simulations offer a sophisticated lens through which to observe and interpret the intricate behaviour of biological molecules at an atomic resolution. In this comprehensive study, MD simulations were executed with the primary intent to rigorously evaluate the structural stability and binding convergence of the complex comprised of 1VPP and OCTADECANOIC ACID. During the extensive 100‐ns simulation period, the 1VPP + OCTADECANOIC ACID complex demonstrated noteworthy conformational stability. This stability was quantitatively assessed through the Root Mean Square Deviation (RMSD) of the Cα‐backbone, which stabilized around an average of 1.6 Å, as delineated in Figure 3A which is a stable curve. Such consistent RMSD values throughout the simulation underscore the reliability of the binding mode and its persistent maintenance over time.

Delving deeper into the dynamics of the protein‐octadecanoic acid interaction, the Root Mean Square Fluctuations (RMSF) were scrutinized. While the overall RMSF plot (Figure 3B) maintained a uniform profile, elevated fluctuations were discerned at residues 170, 370–377, 620–645, and 785–820. Such region‐specific flexibility often alludes to either the residues' strategic involvement in octadecanoic acid interaction or their inherent structural positioning in more flexible regions such as loops. Contrarily, the minimal fluctuations observed in the majority of the residues speak to the structural rigidity and integrity of the protein when in complex with the OCTADECANOIC ACID.

A pivotal metric in understanding the spatial arrangement and compactness of proteins is the Radius of Gyration (Rg). In this investigation, the Rg value of the 1VPP + OCTADECANOIC ACID complex manifested a modest decline from 31.2 Å to 30.7 Å (Figure 3C). Such a decrement in Rg is indicative of a protein adopting a more compact and, potentially, a more energetically favourable conformation upon octadecanoic acid binding.

The establishment and maintenance of hydrogen bonds between a protein and its associated octadecanoic acid often serve as robust indicators of interaction strength and stability. In the context of our study, the hydrogen bond analysis revealed that the 1VPP and OCTADECANOIC ACID complex maintained an average of 1 hydrogen bonding throughout the 100‐ns duration, mirroring the behaviour observed with a standard octadecanoic acid (Figure 3D). Such consistency in hydrogen bond formation not only accentuates the robustness of the octadecanoic acid‐protein interaction but also positions OCTADECANOIC ACID as a binding partner of significant potential.

**FIGURE 4:**
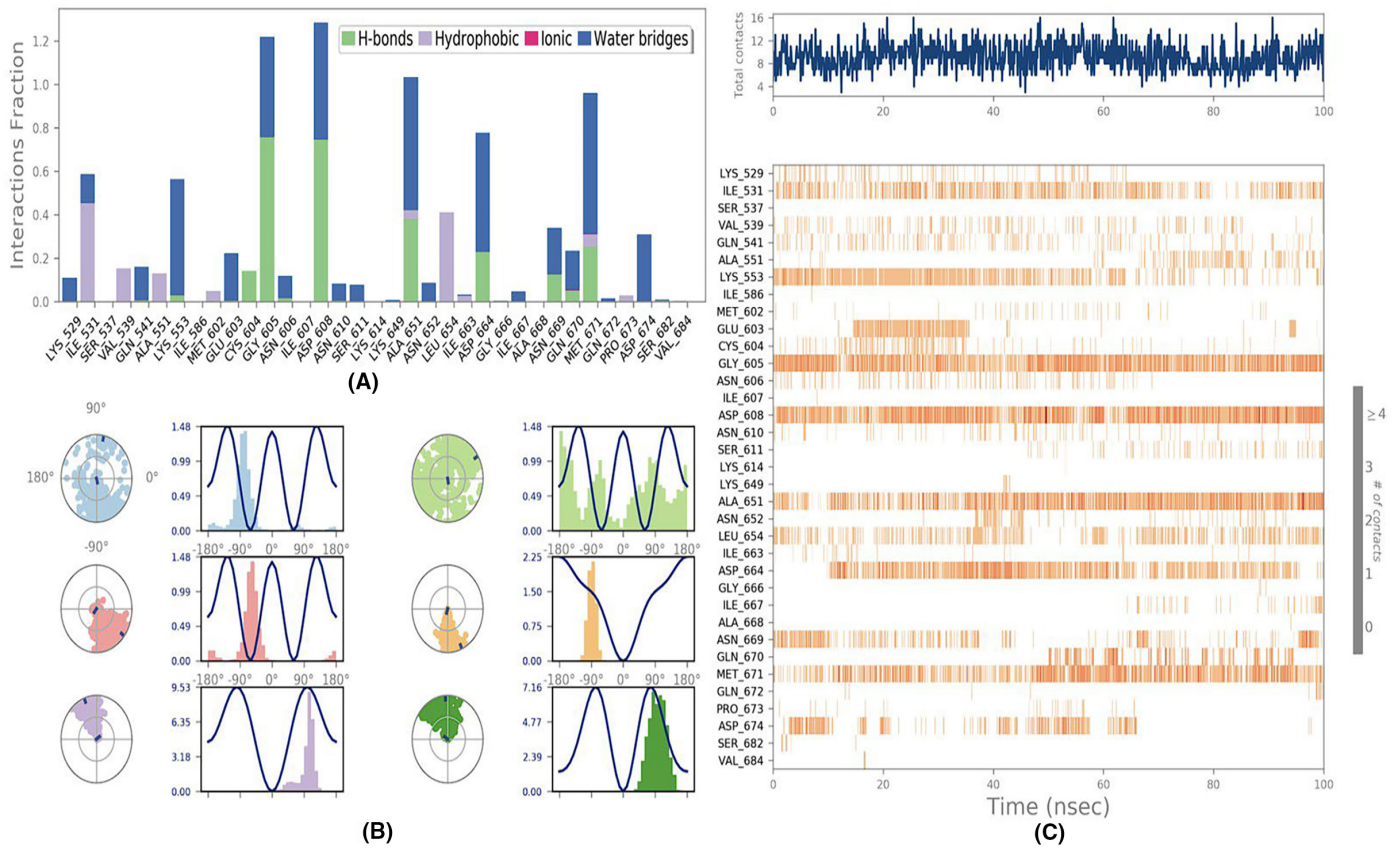
(A) Interaction plot; (B) Ligand torsion profile; (C) Protein‐Ligand contacts.

In summation, the meticulous and advanced MD simulations conducted herein provide a profound understanding of the dynamic interplay between 1VPP and OCTADECANOIC ACID. These revelations hold immense potential for further structural and therapeutic explorations, setting a benchmark in molecular biophysical investigations (Figure 4).

### 3.3 Molecular mechanics generalized born surface area (MM‐GBSA) calculations

Utilizing the MD simulation trajectory, the binding‐free energy along with other contributing energy in form of MM‐GBSA was determined for each complex of 1VPP + OCTADECANOIC ACID. The results (Table 2) suggested that the maximum contribution to Δ*G*_bind_ in the stability of the simulated complexes were due to Δ*G*_bind_Coulomb, Δ*G*_bind_vdW and Δ*G*_bind_Lipo, while, Δ*G*_bind_Covalent and Δ*G*_bind_SolvGB contributed to the instability of the corresponding complexes. These results supported the potential of 1VPP + OCTADECANOIC ACID having high affinity of binding to the protein as well as efficiency in binding to the selected protein and the ability to form stable protein‐octadecanoic acid complexes.

**TABLE 2:** Binding free energy components for the 1VPP + OCTADECANOIC ACID calculated by MM‐GBSA.

Energies (kcal/mol)	1VPP + OCTADECANOIC ACID
Δ*G*_bind_	−54.65 ± 5.79
Δ*G*_bind_Lipo	−16.18 ± 1.04
Δ*G*_bind_vdW	−46.19 ± 2.18
Δ*G*_bind_Coulomb	−14.27 ± 6.20
Δ*G*_bind_H_bond_	−2.93 ± 5.32
Δ*G*_bind_SolvGB	9.34 ± 4.34
Δ*G*_bind_Covalent	6.45 ± 1.51

### 3.4 Anti‐proliferative efficacy of ACE on SiHa cells

**FIGURE 5:**
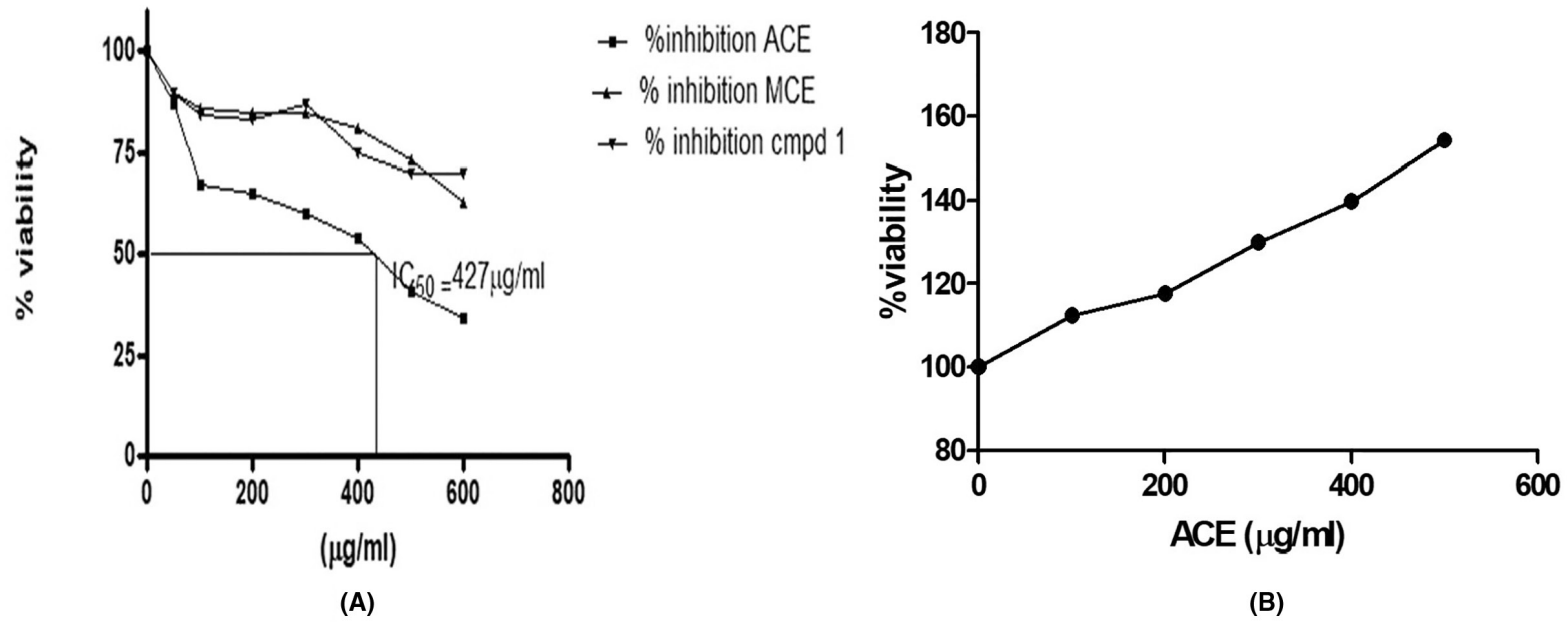
Differential Anti‐Proliferative Activity of ACE, MCE, and Compound 1 on SiHa Cells and PBMCs. (A) Line graph delineating the anti‐proliferative responses of SiHa (HPV16+) cells upon exposure to ACE, MCE, and Compound 1, as determined via the MTT assay; (B) Line graph elucidating the viability profile of healthy peripheral blood mononuclear cells (PBMCs) post‐ACE treatment, as quantified through the MTT assay.

In our meticulous evaluation, the SiHa cells, representative of a cervical cancer cell line, were subjected to the pharmacological influence of the aqueous crude extract (ACE). Upon examination, the findings were compelling. The ACE showcased a pronounced anti‐proliferative stance against these cells, culminating in a noteworthy inhibition rate that peaked at 68.5%. This effect was not merely observational; quantitative assays further solidified the data, pinpointing the IC_50_ value, a pivotal metric in pharmacodynamics, at 427.5 ± 12.5 μg/mL, a detail elegantly plotted in Figure 5A. For comparative analysis, the methanolic crude extract (MCE) was also utilized. With its dissolution in a 0.5% DMSO matrix and spanning concentrations ranging from a modest 100 μg/mL to a more robust 600 μg/mL, the cellular responses were intriguing. A plateau of inhibition was evident at 37.5%, specifically achieved when the concentration touched 500 μg/mL. This difference between the efficacies of ACE and MCE underscores the inherent complexities and nuances in their respective biochemical compositions and their interactions with the cellular environment of SiHa cells. Upon further fractionation and isolation procedures from the ACE, a compound, provisionally termed ‘compound 1’, was derived. Contrary to expectations, this isolated entity exhibited a lacklustre performance in terms of anti‐proliferative activity. Such an outcome beckons deeper investigative endeavours, hypothesizing that the overarching efficacy of ACE might not be due to singular compounds but potentially a synergistic interplay among its constituents.

### 3.5 Differential and selective proclivity of ACE toward healthy PBMC cells

Translational medicine mandates a balanced approach where therapeutic interventions target pathogenic entities without detriment to the surrounding physiological milieu. Anchoring on this doctrine, the ACE's interaction with healthy peripheral blood mononuclear cells (PBMC) was scrutinized. Contrary to potential assumptions of cytotoxicity, ACE manifested an intriguing profile. Instead of hindering the PBMCs, ACE instigated a robust proliferative response, propelling these cells into a heightened state of activity. This profound cellular stimulation is captured in Figure 5B, delineating the extract's selective cytotoxicity, a trait immensely valuable in oncological therapeutics. To encapsulate, the data encapsulated herein serves as a harbinger, extolling the virtues of ACE as a potential luminary in the annals of cervical cancer therapeutics. Not only does it exhibit a targeted assault on cancerous cells, but its benign, if not beneficial, interaction with healthy PBMCs augments its therapeutic potential. As we venture further, a deeper understanding of the molecular mechanics and pathways influenced by ACE will undoubtedly shape the trajectory of targeted, efficacious cancer therapies.

### 3.6 Examination of ACE's impact on cervical cancer cell motility

The intricate process of tumour metastasis is underpinned by the motility of cancer cells. To halt or significantly retard the advancement of cancer, it is crucial to apprehend and target mechanisms that facilitate tumour cell movement. Within this framework, the effect of aqueous crude extract (ACE) on the migratory propensity of cervical cancer cells emerged as a compelling area of investigation.

The chosen modality to elucidate this phenomenon was the wound healing assay. This assay provides an elegant yet robust platform to quantitatively assess cellular migration in a controlled environment. Cervical cancer cells were meticulously cultured under stringent conditions, ensuring their vitality and responsiveness. The IC_50_ dose of ACE, a concentration derived from rigorous prior cytotoxicity evaluations, was administered to the cell culture. The essence of using the IC_50_ dosage was twofold: It presented a scenario where the biological influence of ACE was maximal, yet without veering into overt cytotoxic territory, thus allowing for an undistorted appraisal of cell motility.

**FIGURE 6:**
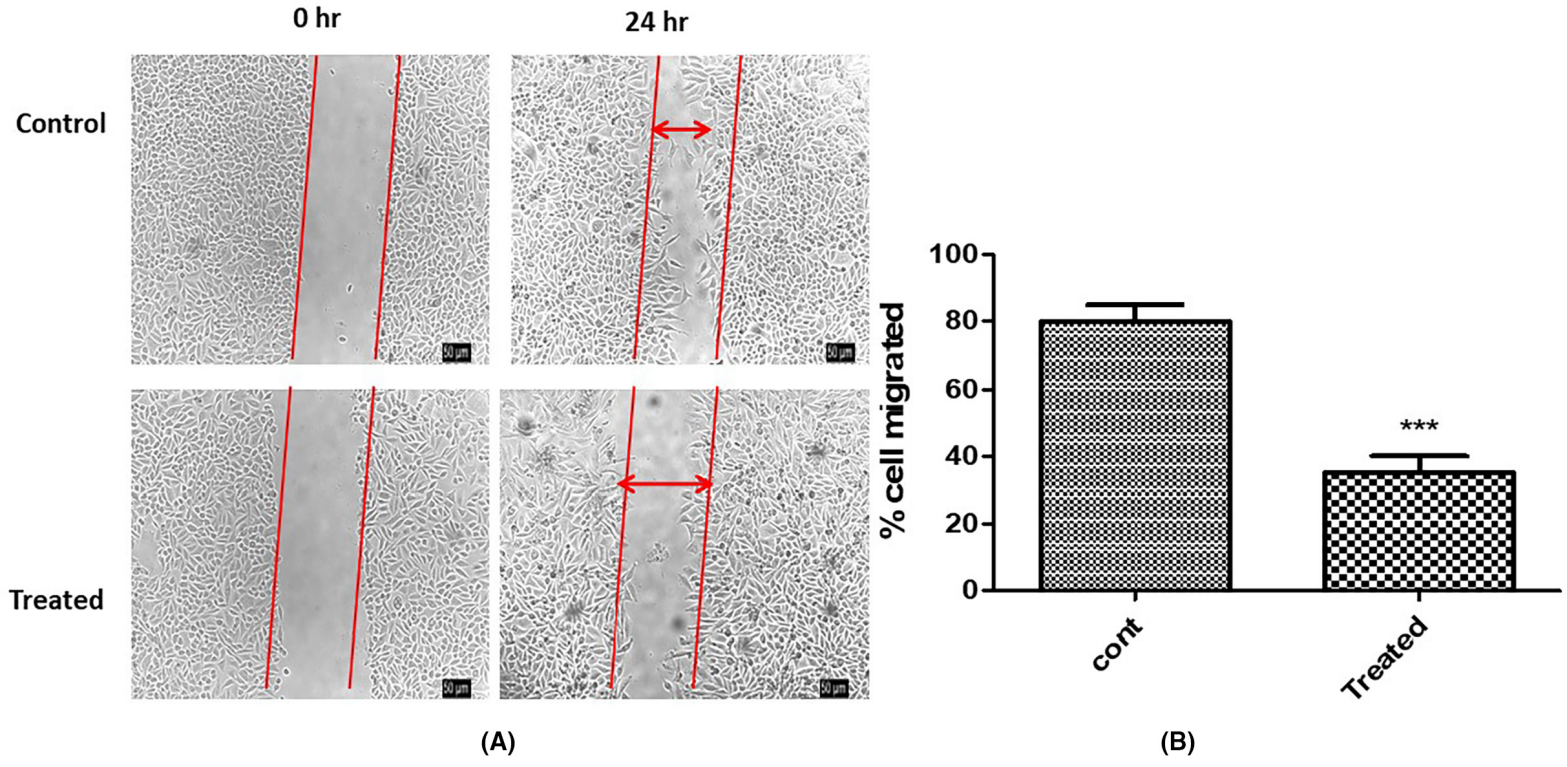
(A) Phase contrast microscopic image of wound healing assay, (B) Bar graph showing percentage of cell migration in wound healing assay (***p < 0.001).

Post a 24‐h incubation period, the treated cells were keenly observed under an advanced inverted microscopy system. Preliminary visual assessments indicated a stark divergence in migratory behaviour between treated and untreated cohorts. Quantitative metrics provided a sharper perspective, revealing a diminution in migration by an arresting 50% in ACE‐treated cells vis‐à‐vis their untreated counterparts. This contrast is microscopically and graphically represented in Figure 6A,B (untreated baseline and post‐ACE treatment migration metrics) respectively. ACE of *C.indica* was found to contain oligosaccharides, fatty acid methyl ester along with aminoglycosides, carboxylic acids, amide esters, which synergistically inhibited the cancer cell migration. These findings bequeath profound implications. The data accentuates that ACE's influence isn't merely restricted to inducing cytotoxic effects on malignant cells. Rather, it harbours the potential to strategically impede their migratory faculties. Such an attribute is of paramount therapeutic importance; curbing metastatic propensities can substantially bolster clinical outcomes. In delving deep into the multifarious impacts of ACE on cervical cancer cells, this study underscores its dual potency: as an inhibitor of cell proliferation and a deterrent to cell migration. As the broader scientific milieu pivots toward a more granular understanding of ACE's molecular interplays, there is burgeoning optimism surrounding its prospective role in oncological interventions.

### 3.7 Assessment of ACE's impact on the clonogenic potential of SiHa (HPV16+) cells

The clonogenic assay, also termed as the colony formation assay, stands as a gold standard in cancer biology, offering profound insights into the ability of single cells to undergo indefinite division and form colonies. This assay effectively unravels the resilience of tumour cells post‐treatment, providing an in‐depth perspective on their proliferative capacity. In this investigative endeavour, SiHa cells, characterized by HPV16+ (Human Papillomavirus type 16) profile, were employed as the model system. To ensure a methodical and rigorous examination, the cells were treated with the IC_50_ dosage of aqueous crude extract (ACE)—a concentration meticulously derived to ascertain significant biological effects without inducing complete cytotoxicity.

Subsequent to the ACE treatment, cells were granted an extended incubation period of 14 days. This duration was judiciously chosen to allow cells ample time to manifest their clonogenic potential, thus facilitating a comprehensive assessment of ACE's long‐term effects.

**FIGURE 7:**
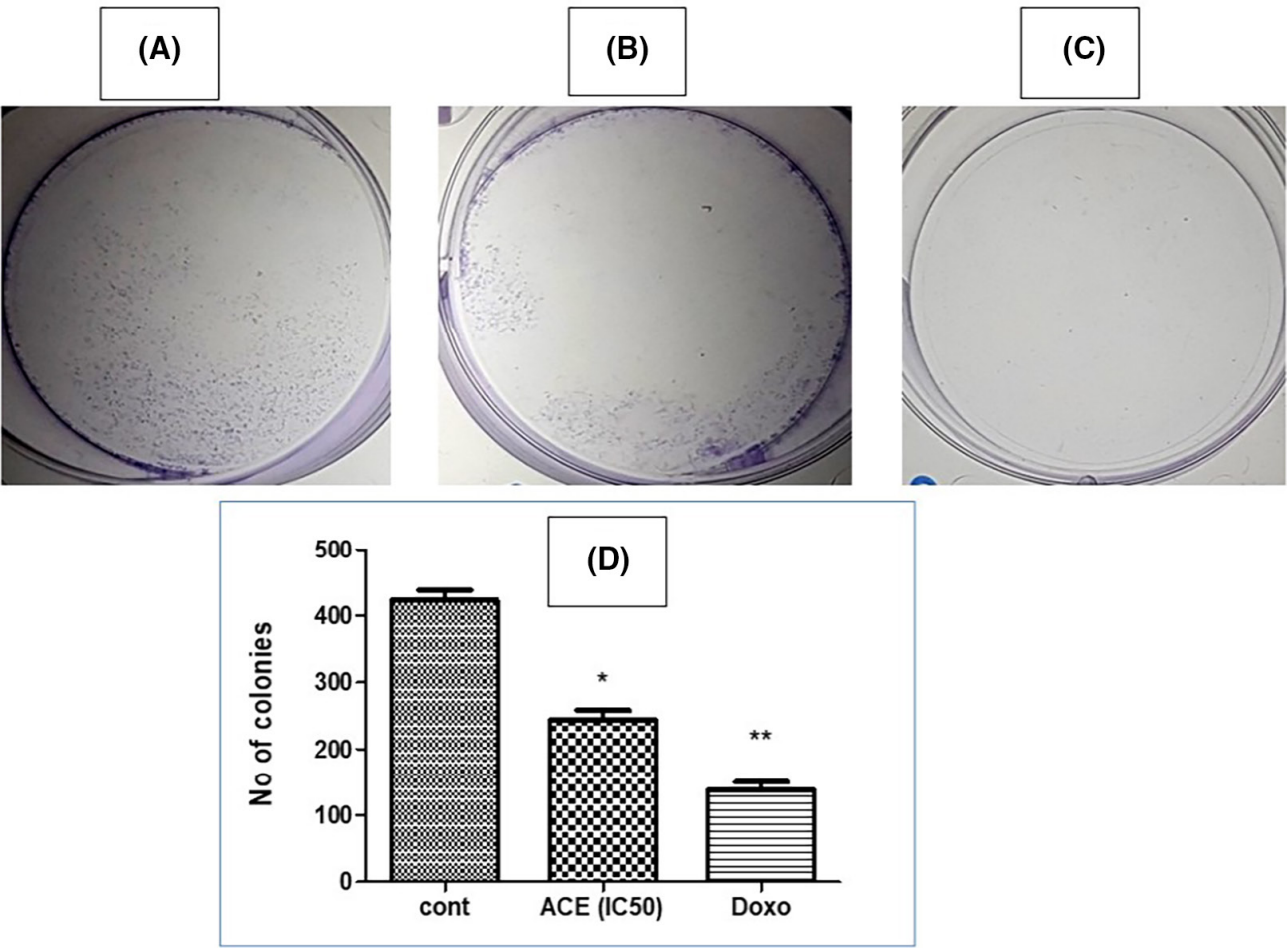
Plates showing colonies of cancer cells after crystal violet staining. (A) control, (B) ACE (IC_50_) treated, (C) Doxorubicin treated, (D) bar graph showing number of colonies in control and treated plates counted by ImageJ software.

The culmination of this incubation period was marked by an examination of the cell plates. To enhance visibility and contrast, colonies were stained using crystal violet, a widely regarded staining agent renowned for its efficacy in demarcating cell colonies. As evidenced in Figure 7, a compelling revelation awaited. The representative plates vividly depicted a striking reduction in colony formation; the treated plates showcased a colony count plummeting to approximately 50% relative to the untreated control group. The tangible reduction in colony formation post‐ACE treatment indicates more than a mere transient suppression of SiHa cell proliferation. It resonates with the potential of ACE to inflict lasting cellular changes, impeding the very capability of these cells to regenerate and establish colonies. This not only underscores ACE's cytostatic properties but also emphasizes its potential as a formidable candidate for therapeutic interventions, especially for conditions orchestrated by hyper‐proliferative cells. The clonogenic assay, as presented, offers an enlightening glimpse into the multi‐faceted capabilities of ACE. By curtailing the proliferative and clonogenic vigour of SiHa (HPV16+) cells, ACE holds promise as an agent of therapeutic relevance, warranting further exploration and validation in diverse oncological contexts.

### 3.8 Evaluation of ACE's influence on ROS generation in SiHa (HPV16+) cells

Reactive Oxygen Species (ROS) are renowned for their double‐edged nature in cellular biology. While they play pivotal roles in cellular signalling and homeostasis, an abnormal surge can lead to oxidative stress, culminating in potential cellular damage or apoptosis. SiHa cells were treated with the IC_50_ dosage of the aqueous crude extract (ACE). The IC_50_ dosage signifies a concentration that elicits a halfway maximal inhibitory response, thereby offering a balanced paradigm to decipher any ROS modulations. *N*‐acetyl cysteine (NAC), a widely‐acknowledged antioxidant, was incorporated as the negative control in this study, setting the baseline against which any ROS alterations could be juxtaposed. Upon treatment, the SiHa cells exhibited a modest decline of 15.5% in ROS levels post‐ACE exposure, as discerned in Table 3. While this decrease is notable, it failed to reach the benchmark of statistical significance. In contrast, NAC's introduction into the experimental milieu led to a 26.8% decrement in ROS generation, underscoring its robust antioxidative credentials. However, the subsequent amalgamation of both NAC and ACE, envisaged to synergistically modulate ROS, culminated in a 23.1% reduction—a value that, while being appreciable, still hovered within the bounds of non‐significance. The presented results weave a narrative of ACE's limited influence on ROS dynamics within SiHa (HPV16+) cells. The relative stability of ROS levels, even in the presence of ACE, delineates a pivotal conclusion: the cytotoxic prowess of ACE does not hinge upon ROS modulation. Such an understanding illuminates alternative mechanisms through which ACE might be orchestrating its cellular impact, laying the groundwork for subsequent investigative probes. Given the intriguing revelation that ACE's cytotoxicity isn't ROS‐dependent, it is prudent to delve deeper into the molecular underpinnings governing ACE's interactions with SiHa cells. Such pursuits can unlock newer mechanistic insights, further solidifying or potentially diversifying the therapeutic potential of ACE in oncological applications.

**TABLE 3:** Evaluation of ROS generation in SiHa cells.

ACE (IC50)	NAC	NAC + ACE
% ROS decrease		
15.5	26.8	23.1

### 3.9 Cell cycle analysis

**FIGURE 8:**
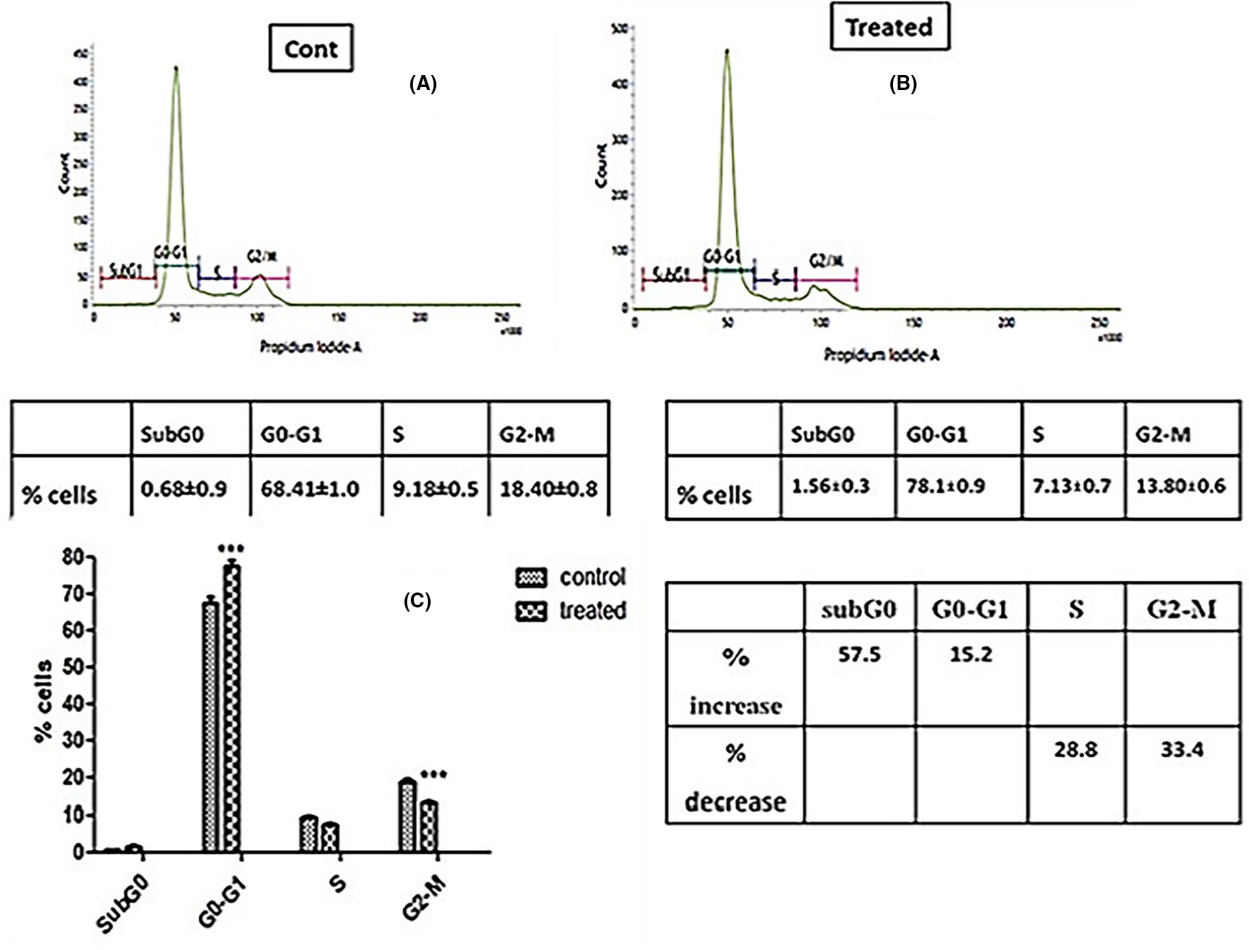
(A) Histogram showing cell cycle distribution of control cells, (B) Histogram showing cell cycle distribution of treated cells with IC_50_ dose of ACE, (C) Bar graph showing variation of cell cycle distribution in control and treated (***p < 0.001).

The cell cycle is a regulated sequence of events leading to cell division and DNA replication, critical for cell growth and proliferation. Aberrant cell cycle progression is a hallmark of many cancers, making it a prime target for therapeutic interventions. Evaluating the impact of external agents on cell cycle distribution can offer insights into their mode of action and potential anticancer properties. The percentage of SiHa (HPV16+) cells in different phases of cell cycle were analysed by flow cytometry. IC_50_ dose of ACE significantly increased the proportion of cells in G0‐G1 phase by 15.2% with a decrease in S and G2‐M phase by 28.8% and 33.4% respectively compared to control (Figure 8). Increase in subG0 popoluation was not significant which indicate the extract arrested the cell cycle progression at G1phase and acting as a cytostatic agent.

### 3.10 Apoptosis assay (annexin V‐FITC/PI double staining)

**FIGURE 9:**
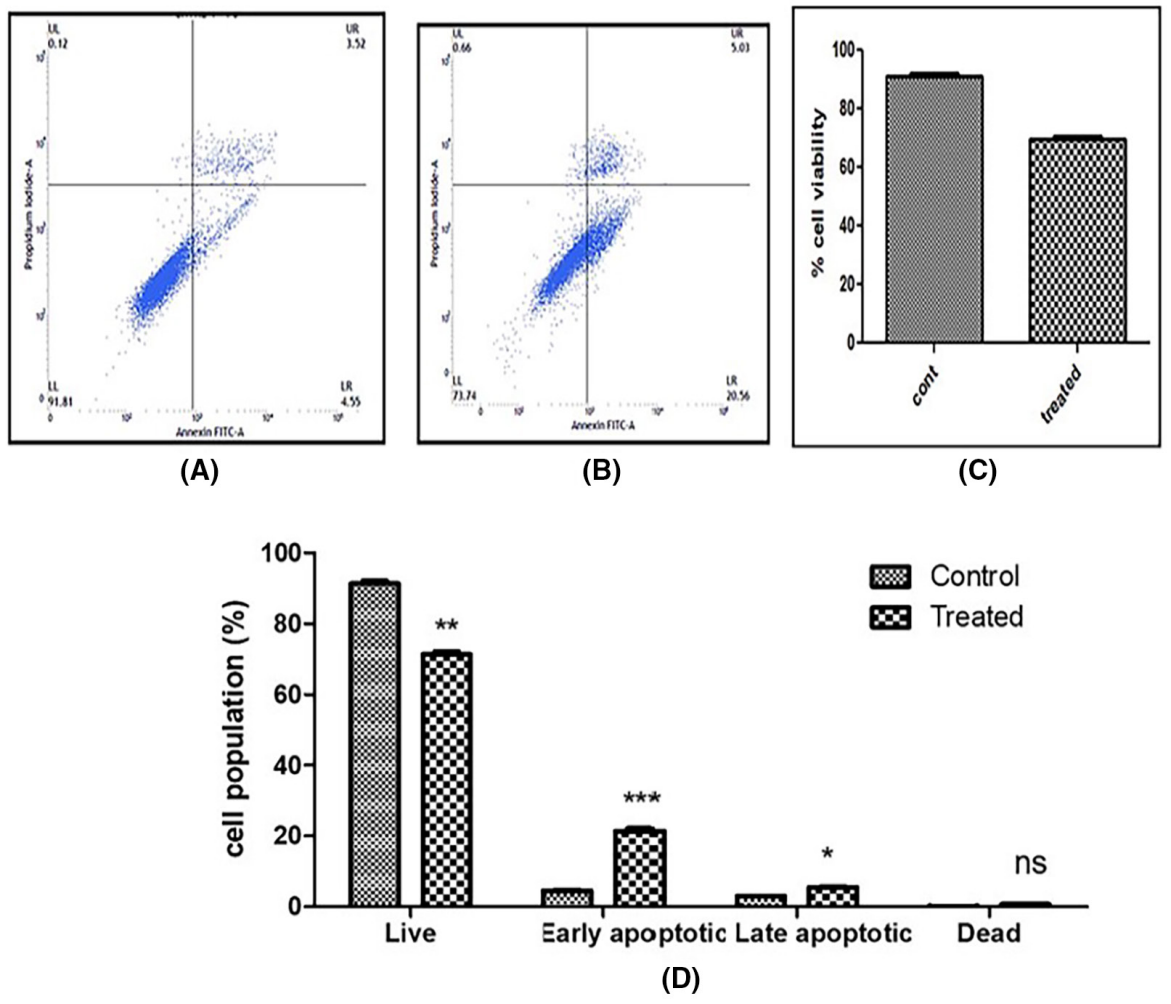
(A, B) Representative dot plots of control and treated cells, (C, D) Cell distribution in different sets of SiHa cells after Annexin V‐FITC/PI staining is shown as a bar graph; column shows cell populations, while bars reflect standard deviations. (Ns, not significant, *p < 0.05, **p < 0.01, ***p < 0.001).

In SiHa cells, only 8.07% early and late apoptotic cells were found in control cells, whereas ACE (IC50)‐treated SiHa cells showed threefold increase in cell death with 25.59% early and late apoptotic cells (Figure 9).

### 3.11 Nuclear morphology

**FIGURE 10:**
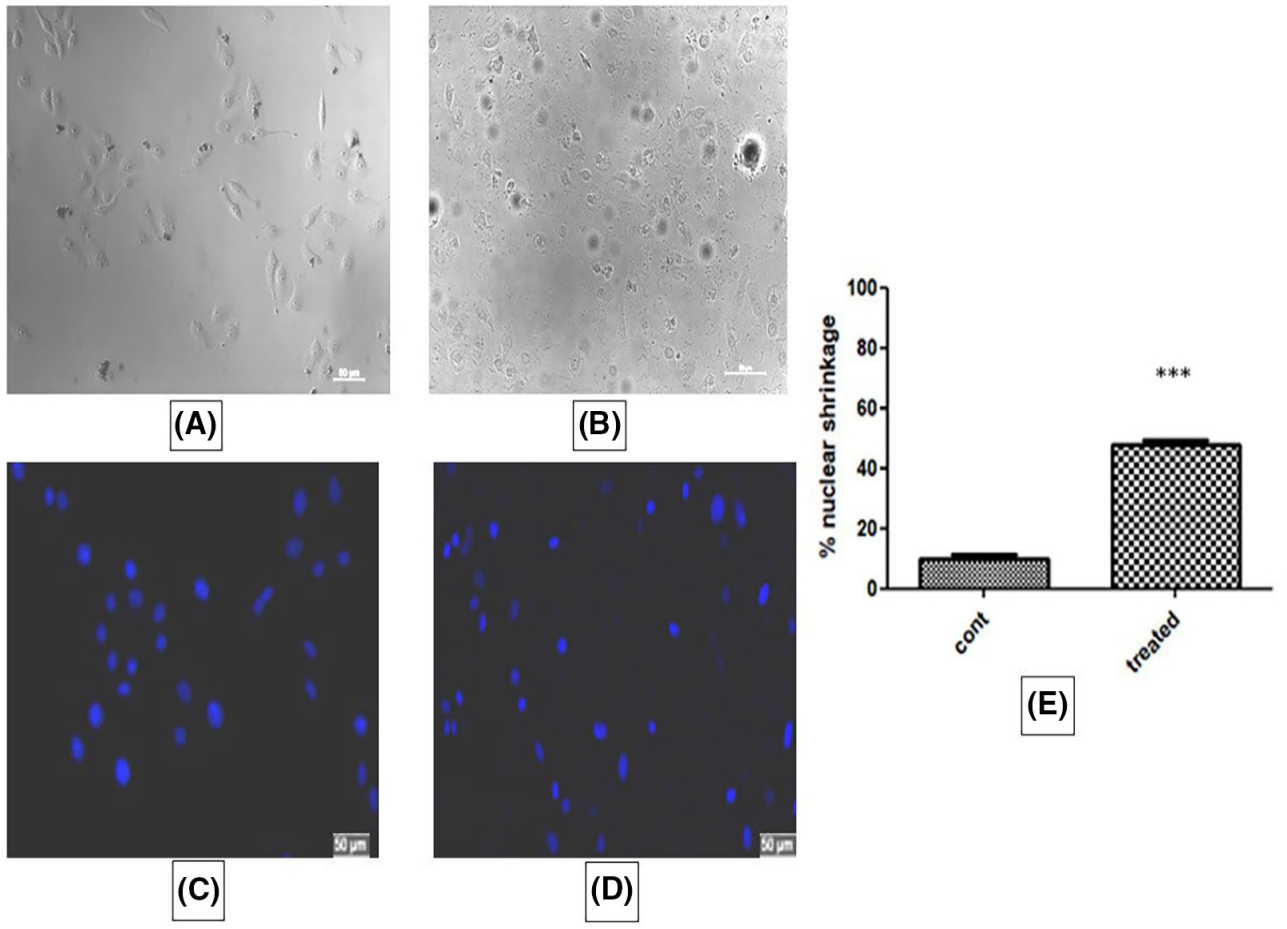
Change in nuclear morphology of SiHa cells. (A, C) Phase contrast and fluorescence image SiHa (HPV16+) control cells, (B, D) In comparison to control cells, phase contrast and fluorescence images of ACE induced changes in nuclear morphology in SiHa cells. The scale bar is 50 μm long, and the yellow arrows point to the deeply condensed shrivelled nuclei. (E) Bar graph showing % of nuclear shrinkage in treated cells compared to control (***p < 0.001).

In treated cells, changes in nuclear morphology were observed to some extent. Membrane shrinkage and cytoplasmic blebbing's were not significant. Hoechst staining showed condensation of nucleus in almost 50% of cells treated with IC_50_ dose of ACE compared to control which indicates early apoptosis (Figure 10).

### 3.12 Characterization of *C. indica* aqueous crude polysaccharides (ACP)

The percentage yield and biochemical characterization with respect to total sugar, total reducing sugar, total ß‐glucan content of aqueous crude polysaccharide (ACP) isolated from aqueous crude extracts (ACE) of *C. indica* are shown in Table 4.

**TABLE 4:** Characterization of *C. indica* aqueous crude extract (ACE) and aqueous crude polysaccharide (ACP).

Total ACE yield, mg/100 mg of dry fruiting bodies	Total ACP yield, mg/100 mg of ACE	Total polysaccharide content, mg/100 mg of ACP	Total reducing sugar content, mg/100 mg of total polysaccharides	Total ß‐glucan content, mg/100 mg of total polysaccharides
2.5 ± 0.5	0.5 ± 0.02	20.0 ± 3.20	11.5 ± 2.95	1.2 ± 0.6

### 3.13 Growth stimulation activity of *C. indica* polysaccharides (ACP) on *Lactobacillus* spp.

**FIGURE 11:**
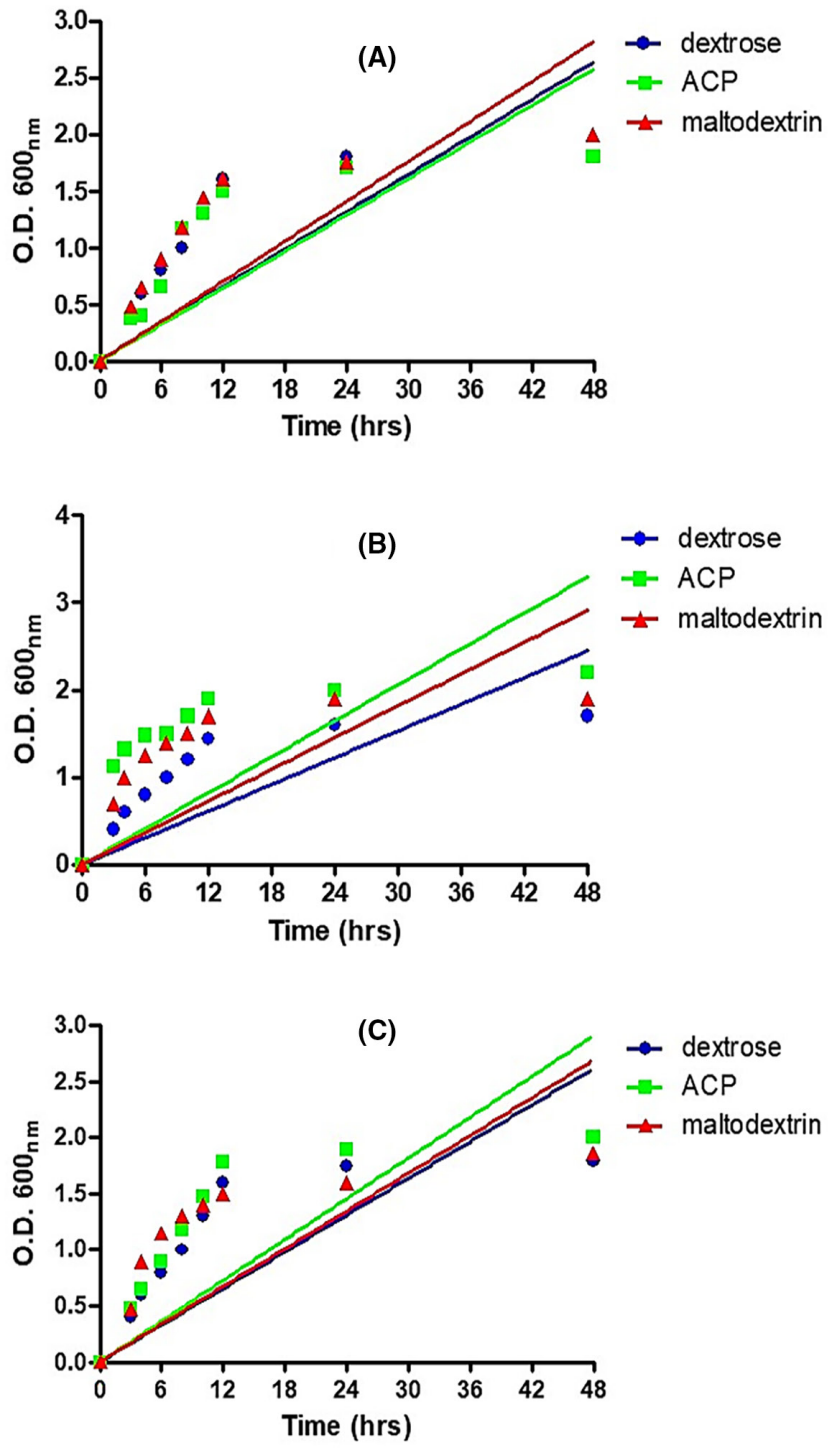
Growth stimulation activity of dextrose, maltodextrin and aqueous crude polysaccharides (ACP) on *Lactobacillus* spp. (A) Lactobacillus plantarum, (B) *Lactobacillus rhamnosus*, (C) *Lactobacillus acidophilus*.

The ability of aqueous crude polysaccharides (ACP) to promote the growth of *Lactobacillus* spp. was determined in vitro by measuring absorbance at different time interval (Figure 11). After 48 h, maximum growth was observed for *L. acidophilus* and *L. rhamnosus*. ACP revealed 12%–15% growth induction compared to dextrose and maltodextrin. However, there was no growth induction for *L. plantarum*. The commercially available prebiotic maltodextrin (Himedia) was used as positive control. It showed 10%–12% of growth induction which was comparable with ACP. There was 10%–15% reduction in pH of culture media 3 when treated with ACP and maltodextrin respectively and 20% reduction in pH for dextrose after 48 h of incubation (Figure 11).

### 3.14 Resistance towards acid digestibility

**FIGURE 12:**
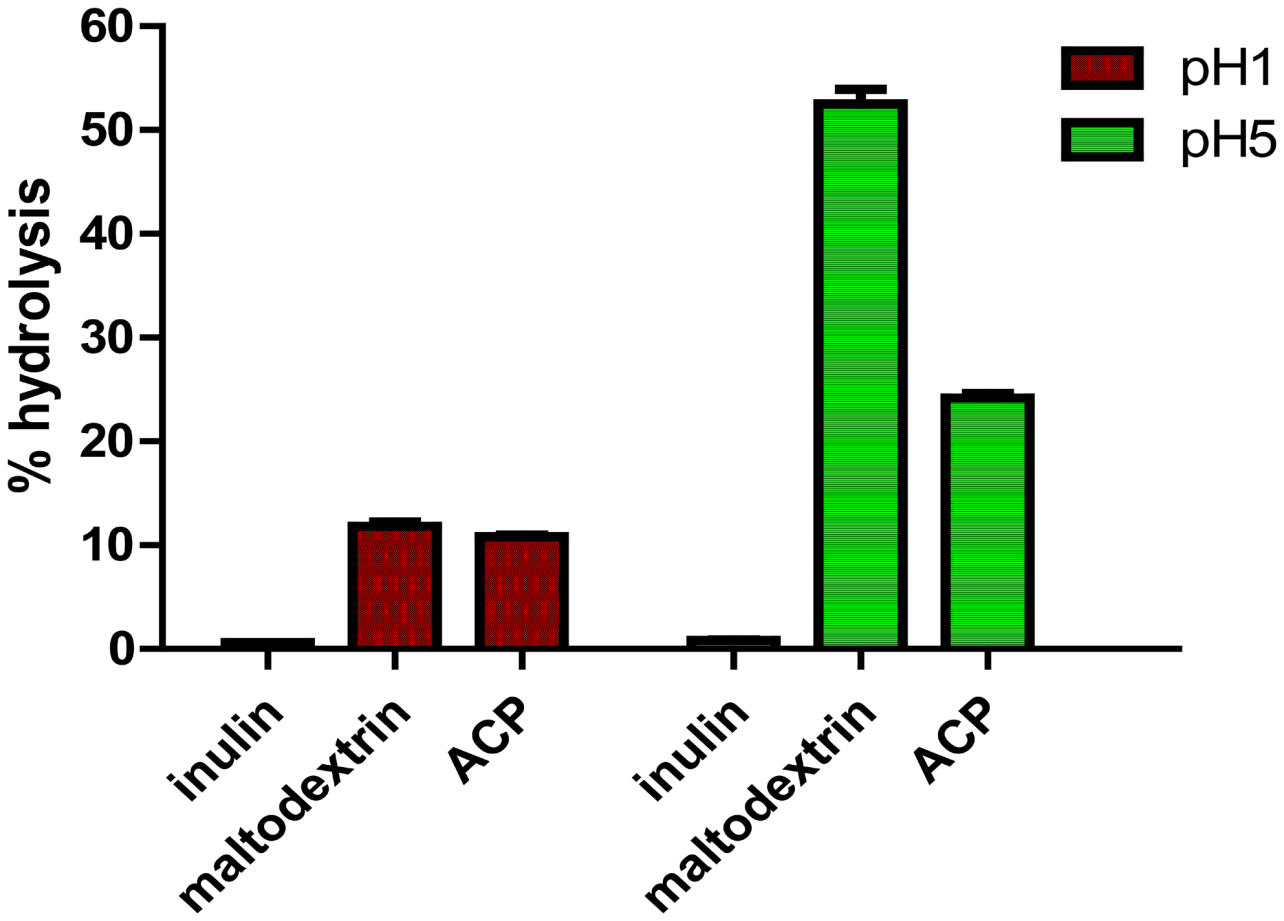
The degree of polysaccharide hydrolysis when exposed to synthetic human gastric juice at pH values ranging from 1 to 5.

The degree of hydrolysis for ACP, inulin and maltodextrin when subjected to artificial gastric juice buffer with pH 1 and pH 5 are shown in Figure 12. The result obtained for pH 1 ranges from 10.86%–11.56% hydrolysis, while for pH 5, the value ranges from 20 to 25% hydrolysis for *C. indica* ACP. The degree of hydrolysis was found to be minimum for inulin with 0.6%–0.8% respectively, but maltodextrin revealed higher degree of hydrolysis than ACP with 12.5% at pH 1 and 50%–55% at pH 5. The mushroom polysaccharide investigated remained more than 90% undigested at pH 1. These findings suggested that polysaccharides can pass through the stomach in their natural form and reach the colon, where they can enhance the growth of beneficial bacteria. The composition and structure of polysaccharides extracted from mushrooms and their possible interactions with some other molecules may clarify the variations in hydrolysis under different pH conditions.

### 3.15 Characterization of ACE by HRLC‐MS study

**FIGURE 13:**
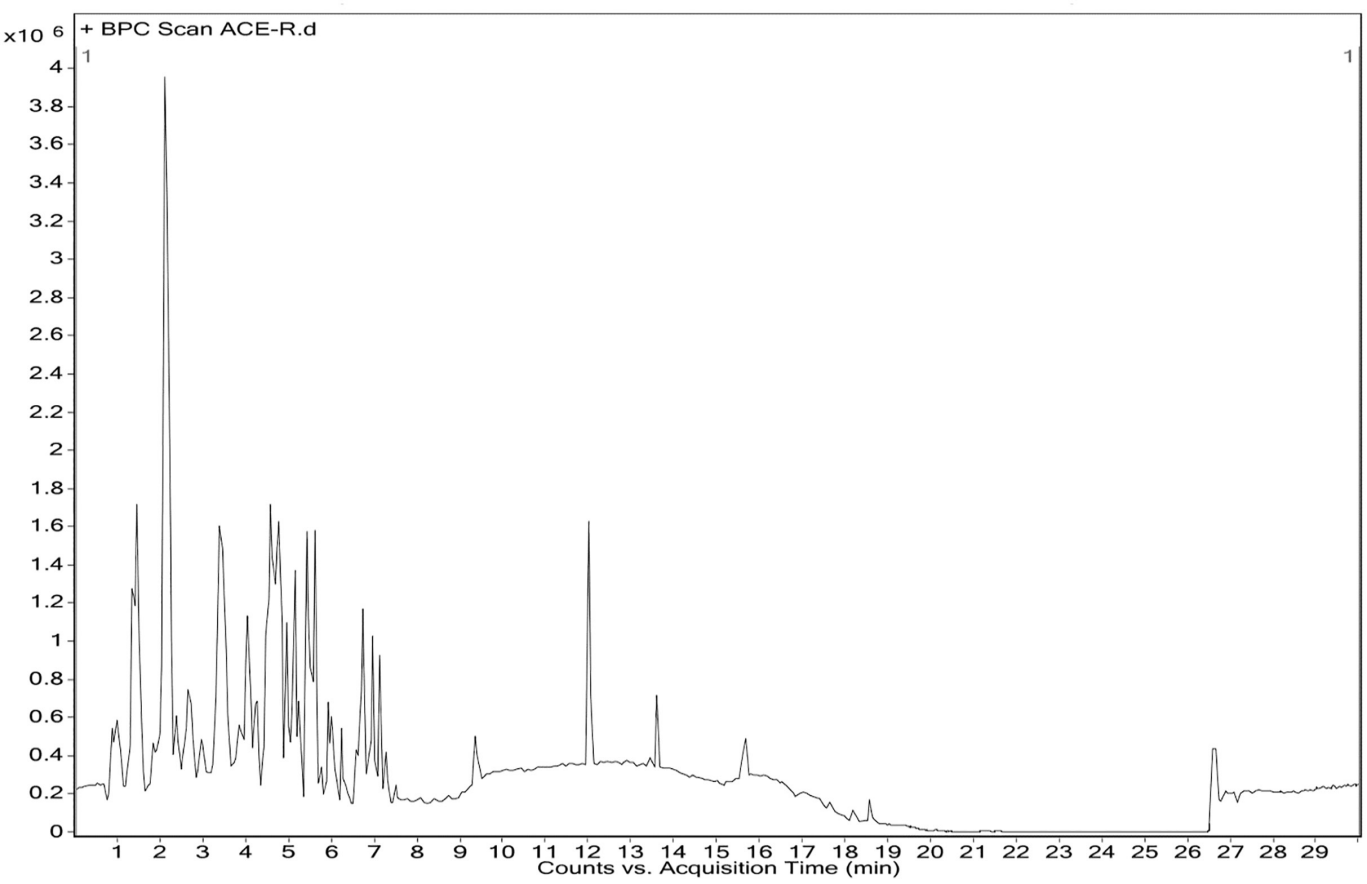
Chromatogram of ACE showing peaks of major bioactive compounds by HR‐LCMS study. Peak 1: maltose, peak 2: 2‐propylisonicotinic acid, peak 3: ziduvodine monophosphate, peak 4: butabarbital, peak 5: altretamine, peak 6: meradimate, peak 7: 3‐amino‐2‐napthoic acid, peak 8: proadifen, peak 9: 10,12,15 octadecatrienoic acid, peak: 10: 9, 13‐dihydroxy‐11‐octadecanoic acid.

HR‐LCMS analysis of aqueous water extract (ACE) of *C. indica* fruiting bodies showed major peaks indicating the presence of various phytochemical constituents (Figure 13). ACE contains oligosaccharides, fatty acid methyl ester along with aminoglycosides, carboxylic acids, amide esters, which have reported anticancer activity (Table 5).

**TABLE 5:** Chemical constituents of ACE studied through HR‐LCMS analysis.

Name	Class	Mass/formula	Relative content	RT
Maltose	Reducing sugar	342.3 C1H22O11	15.95	1.02
Ziduvodine monophosphate	Nucleoside analogue	347.07 C10 H14 N5 O7 P	19.4	1.31
Butabarbital	Amide esters	212.11 C10 H16 N2 O3	8.9	1.42
2‐Propylisonicotinic acid	Pyridine derivative	165.07 C9 H11 N O2	39.5	2.13
Meradimate	Carbocyclic acids	275.18 C17 H25 N O2	7.4	2.52
3 Amino 2‐napthoic acid	Carboxylic acids	187.06 C11 H9 N O2	11.3	4.06
Altretamine	Alkylating agent	210.15 C9 H18 N6	5.62	4.60
Proadifen	Aromatic amines	353.22 C23 H31 N O2	3.5	7.54
10,12,15 Octadecatrienoic acid	Fatty acids	278.22 C18 H30 O2	3.5	12.01

### 3.16 Isolation and identification of compound 1

**FIGURE 14:**
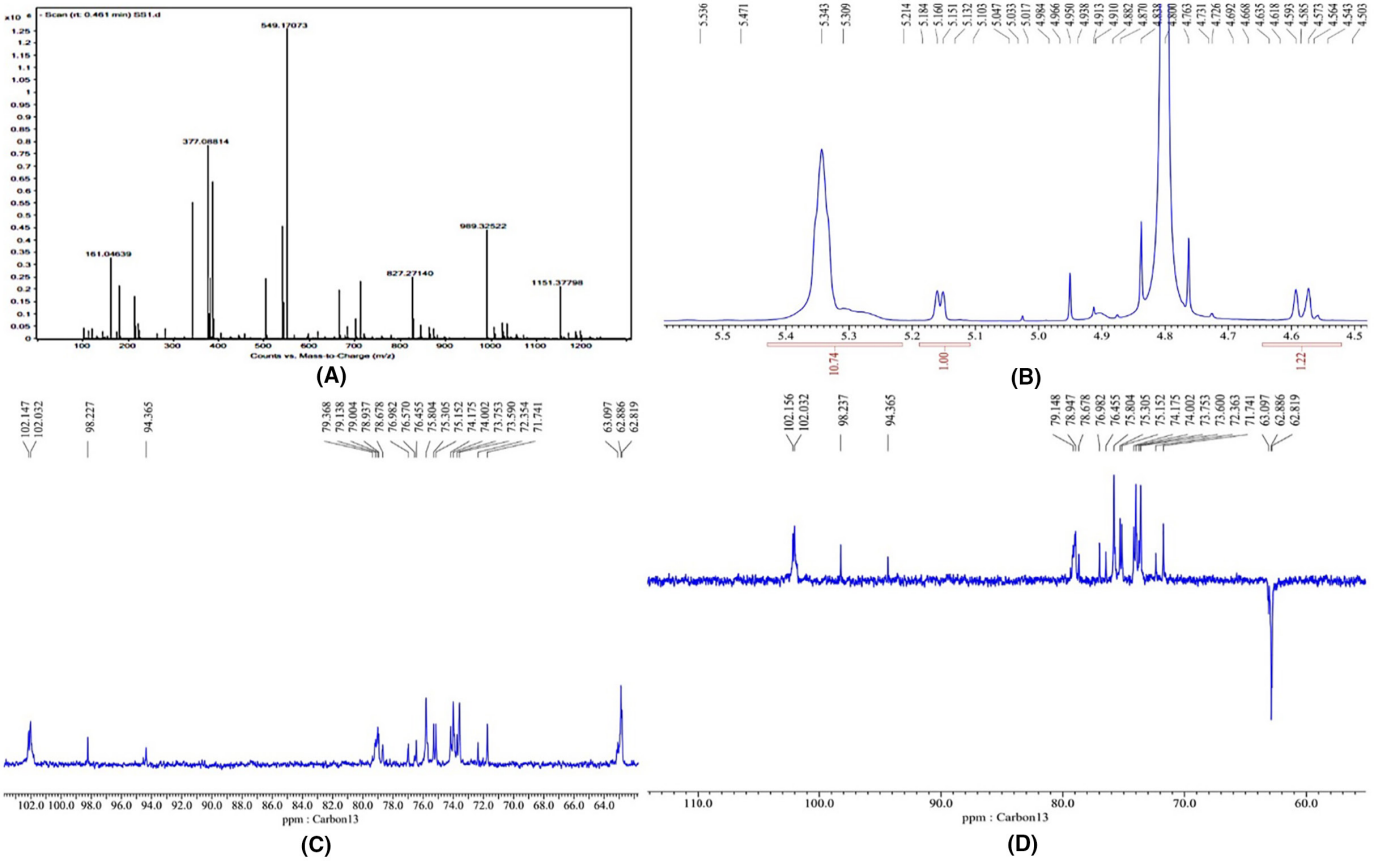
Identification of compound 1: (A) MS of fraction 37–44 identified as a mixed compound, (B) MS of compound 1, (C) 1H NMR of compound 1, (D) 13C NMR of compound 1.

Fractions (1–60) eluted with 0–0.2 M NaCl from Silica gel (60–100, Merck) column were collected and fractions giving similar spots on TLC were combined and purified by chromatography over a column of silica gel (100–200 mesh). The fractions were combined together and crystallized. The crystals obtained were yellowish in colour, transparent, and rhomboidal. The crystals were soluble in water. The compound gave positive Molisch tests suggesting the presence of sugar. The quasi‐molecular ion peak [M + Na] ^+^ appeared at *m*/*z* 342.5 (Figure 14). This information together with 1H NMR and 13C NMR hypothesized to be di‐saccharide in nature with suggested molecular formula to be C_12_H_22_O_11_. Unfortunately, purified compound has no significant bioactivity which indicates the role of synergistic action of the phytochemical compounds present in *C. indica*.

## 4 DISCUSSION

Over the years, the therapeutic potential of various mushrooms in combating cancer has been a focal point of scientific research, drawing attention to their notable anti‐tumorigenic activities across diverse cancer cell lines. Research on the anticancer activity of mushrooms showed substantial anti‐tumorigenic activity on different cancer cell lines both in vivo and in vitro. The mushrooms that showed activity against cervical cancer are *Pleurotus*, *Agaricus*,^34^ *Ganoderma*,^35^ *Cordyceps*,^36^ *Schizophyllum*,^37^ *Inonotus*.^38^ Our research enhances this body of knowledge by exploring the prospective anticancer properties of *C. indica*, a mushroom species which, while previously recognized for its cytotoxic potential against cancer cell lines like HeLa and MCF 731, remains relatively underexplored in broader cancer research.^39^, ^40^

Our investigation specifically centres on its impact on the SiHa (HPV 16+) cervical cancer cell line. One salient discovery of our study is the selective cytotoxicity exhibited by the aqueous crude extract (ACE) derived from *C. indica*. This extract not only revealed potent anti‐cancer activities against SiHa cells but also seemed to favour the proliferation of healthy PBMC cells. Some previous studies reported the hot water and methanolic extracts of *Calocybe indica* potentially inhibited the lung adenocarcinoma cell line and breast cancer cell line in dose dose‐dependent manner.^41^, ^42^ Techniques such as annexin–V‐FITC and PI dual staining, complemented by Hoechst staining, offer strong evidence of ACE‐induced early and late apoptotic shifts. Mandal et al 2011 reported the heteroglycan isolated from a hybrid variety of *C.indica* expressed its cytotoxic potential against human cervical cancer cell line HeLa by apoptosis mechanism.^43^ Beyond its anticancer properties, our study proposes *C. indica's* potential as a source of prebiotic agents, especially given the critical role of *Lactobacillus* in cervical cancer prevention.^44^ Our findings accentuate the capability of *C. indica* polysaccharides to augment the growth of these beneficial bacteria. Leveraging advanced chromatography techniques alongside mass and NMR spectroscopy, we have identified specific phytochemical compounds within *C. indica*, laying the foundation for further exploration of their individual and combined effects. Synthesizing these findings, *C. indica* emerges as a potent dual‐source: as an anticancer agent and a prebiotic powerhouse. The nuanced understanding that ACE hinders cancer cell growth while simultaneously supporting healthy cell proliferation propels *C. indica* to the forefront as a prospective candidate for anticancer therapeutic strategies. The potential synergistic action of the various phytochemical compounds in ACE, even if they don't exhibit significant bioactivity individually, suggests a deeper, integrated mechanism at play. Parallelly, our intricate Molecular Dynamics (MD) simulations provide detailed insights into the nuanced interactions between VEGF and Octadecanoic acid. The consequent analyses from RMSD accentuate the promise Octadecanoic acid holds as an influential binding partner to VEGF. Our findings, derived from the RMSF profiles, demonstrate a commendable equilibrium between structural robustness and protein adaptability. Delving further, the Radius of Gyration studies offer perspectives on how ligand interactions can shape protein conformation. Additionally, our in‐depth MM‐GBSA calculations illuminate the thermodynamic binding landscape of the VEGF and Octadecanoic acid complex. While certain nuances prompt us to consider stability parameters, the overarching narrative from MM‐GBSA underscores the pronounced binding affinity of Octadecanoic acid with VEGF. Emerging therapeutic modalities, including proteolysis‐targeting chimeras (PROTACs) and innovative nanocarriers such as exosomes and liposomes,^28^, ^29^, ^30^ further elevate the therapeutic potential when considered in tandem with compounds like *C. indica*. The integration of these facets suggests a fertile ground for future therapeutic endeavours. This study positions *C. indica* as a vital nexus in the burgeoning field of natural anticancer and prebiotic research. It underscores the need for extended research, particularly in understanding the molecular mechanisms at play, clinical implications, and its role in advancing integrated cancer therapeutics. Such a multidimensional approach promises a richer understanding of *C. indica's* potential roles in both cancer prevention and treatment, ushering in a new era of holistic therapeutic strategies.

## 5 CONCLUSION AND FUTURE PERSPECTIVES

In the dynamic sphere of personalized medicine, the evolution of precision therapeutics is ushering in a novel paradigm in the realm of disease intervention. This modern approach transcends traditional methodologies, embedding itself deeply into the intricacies of genetic sequencing, molecular drug design, and individualized patient care frameworks. The overarching vision shifts from a generalized to a nuanced, evidence‐driven, patient‐centric model of therapeutic intervention. Our comprehensive research on the aqueous crude extract (ACE) of *C. indica* in the context of cervical cancer treatment is emblematic of this shift. We observed that ACE not only influences nuclear morphology but also demonstrates the potential to modulate the cell cycle, instigating early apoptosis and exhibiting prebiotic properties, thus marrying age‐old botanical knowledge with contemporary medical innovationsIn summation, our research artfully blends the tenets of precision therapeutics, the multifarious potentials of *C. indica*, and the intricate dance of molecular interactions between VEGF and Octadecanoic acid. As we navigate this scientific tapestry, our focus remains steadfast on distilling these insights into actionable clinical applications with profound ramifications for holistic patient care. Our journey stands as a testament to the promising confluence of time‐honoured knowledge and modern scientific rigour.

## 6 LIMITATIONS OF THE STUDY

In the diligent pursuit of understanding the therapeutic potential of *C. indica*, this study has shed light on several promising avenues. However, as with all scientific inquiries, certain aspects provide room for deeper exploration. Primarily, our findings revolve around the cervical cancer cell line SiHa (HPV 16+), suggesting that a more comprehensive assessment across diverse cancer cell lines might elucidate the broader anticancer properties of *C. indica*. Additionally, while the study successfully highlighted early apoptotic features, the nuances of late apoptotic mechanisms remain relatively less explored, calling for a more intricate mechanistic dissection in subsequent research. Our identification of specific phytochemical compounds within the ACE of *C. indica* is certainly promising, but a holistic phytochemical profiling could unearth further bioactive constituents with potential therapeutic significance. It is also imperative to note that our in vitro findings, while ground‐breaking, necessitate validation through rigorous in vivo studies to ascertain physiological implications and potential side effects in a holistic organism environment. This research also underscores the importance of delving deeper into the dose–response relationship of the extract, aiming to discern its therapeutic window and optimal efficacy. Lastly, considering the potential of *C. indica* as a prebiotic agent bolstering *Lactobacillus spp*, we recognize the need for extended studies to fathom the long‐term implications of these effects. As we move forward, these considerations will serve as pivotal touchstones, guiding our quest to harness the full therapeutic promise of *C. indica*.

## AUTHOR CONTRIBUTIONS

**Suhana Datta:** Conceptualization (equal); data curation (equal); formal analysis (equal); investigation (equal); methodology (equal); visualization (equal); writing – original draft (equal). **Preeti Verma:** Data curation (equal); formal analysis (equal); investigation (equal); methodology (equal); validation (equal); writing – original draft (equal). **Bikram Dhara:** Conceptualization (equal); data curation (equal); formal analysis (equal); methodology (equal); software (equal); supervision (equal); visualization (equal); writing – original draft (equal); writing – review and editing (equal). **Rita Kundu:** Conceptualization (equal); investigation (equal); project administration (equal); supervision (equal); validation (equal); visualization (equal); writing – review and editing (equal). **Swastika Maitra:** Investigation (equal); methodology (equal); validation (equal); writing – review and editing (equal). **Arup Kumar Mitra:** Conceptualization (equal); investigation (equal); project administration (equal); resources (equal); supervision (equal); validation (equal); visualization (equal); writing – review and editing (equal). **Mohd Shahnawaz Khan:** Formal analysis (equal); investigation (equal); methodology (equal); software (equal); validation (equal); writing – review and editing (equal). **Torki A. Zughaibi:** Formal analysis (equal); investigation (equal); methodology (equal); resources (equal); software (equal); writing – review and editing (equal). **Shams Tabrez:** Investigation (equal); methodology (equal); project administration (equal); supervision (equal); validation (equal); writing – review and editing (equal). **Ajoy Kumer:** Project administration (equal); supervision (equal); visualization (equal).

## CONFLICT OF INTEREST STATEMENT

The authors declares no conflict of interrest exists.

## Data Availability

All the relevant data have been included in the article.
